# Diachronic Analysis of the Floristic Diversity of the Special Area of Conservation (SAC) “Bosco di Santo Pietro” (South-Eastern Sicily): A Mediterranean Biodiversity Hotspot

**DOI:** 10.3390/plants14050788

**Published:** 2025-03-04

**Authors:** Dario Azzaro, Salvatore Cambria, Manuela Porrovecchio, Pietro Minissale

**Affiliations:** Department of Biological, Geological and Environmental Sciences, University of Catania, Via A. Longo 19, 95125 Catania, Italy; azzaro.dario@gmail.com (D.A.); p.minissale@unict.it (P.M.)

**Keywords:** alien plants, conservation, endemic plants, flora, Natura 2000 network, rare plants, red list

## Abstract

This study presents a complete and updated checklist of the vascular flora of the SAC “Bosco di Santo Pietro”, an important natural area located in south-eastern Sicily. Through an integrated approach combining field research, analysis of historical herbariums and a literature review, 583 taxa belonging to 78 families and 339 genera were identified. A comparison with an older floristic list of the Santo Pietro Forest dating back to 1889 highlights some significant changes, such as a decrease in several hygrophilous and nemoral species probably due to climate change and habitat degradation. In particular, our diachronic analysis reveals the disappearance of 178 taxa and the persistence of 199 taxa representing 47% and 53% of the 377 taxa listed in the first inventory, respectively. From the study of the Ellenberg indicator of the two lists, lower values of L and T and higher values of M and N emerge in the older flora, testifying to the significant environmental modifications for more than a century of this Sicilian and Mediterranean hotspot.

## 1. Introduction

Vascular flora represents a fundamental element in the characterization of terrestrial ecosystems, providing crucial information on the biodiversity, structure and functioning of habitats. In particular, the Mediterranean area is recognized as a global hotspot of plant biodiversity, hosting a rich variety of species, many of which are endemic or rare [1,2]. The elaboration of updated and detailed floristic checklists is of fundamental importance because they provide an essential knowledge base for future ecological and biogeographical research [3,4]. They allow us to assess the conservation status of an ecosystem and monitor changes over time. They also support the development of effective management and conservation strategies and contribute to the understanding of the distribution patterns of plant species in the Mediterranean area [5,6,7,8]. In this context, the study of the flora of the Bosco di Santo Pietro, one of the areas with the greatest biodiversity in Sicily [9], has a significant importance also in consideration of the rapid ecological changes that are taking place across the earth.

The woodland of Santo Pietro, located in south-eastern Sicily, represents an ecosystem of considerable floristic and biogeographical interest. From a phytogeographical point of view, it belongs to the Camarino–Pachinense District, one of the smallest but floristically peculiar areas of Sicily [10]. Despite its importance, a complete and up-to-date checklist of its vascular flora is still lacking. Previous specific studies, such as that of Taranto and Gerbini [11] and Furnari [12] provided partial and non-exhaustive information on the floristic composition of the area. Other more recent contributions on this area concern only floristic notes on some species of phytogeographic interest and specific groups such as the Orchidaceae [13,14,15,16,17,18,19]. Conversely, a rather significant list is provided by Zambrano [20], who listed 377 taxa for the area. This study therefore aims to fill this gap, providing a complete and updated checklist of the vascular flora of the Santo Pietro woodland. At the same time, the comparison with the floristic data reported by the abovementioned author allows us to carry out a diachronic analysis of the flora and to highlight the changes that have occurred over more than a century. The ultimate goal is to understand the causes of the observed changes in vascular flora, providing a reference tool for management bodies and contributing to the conservation and enhancement in this important Sicilian natural heritage.

## 2. Results

**Figure 1:**
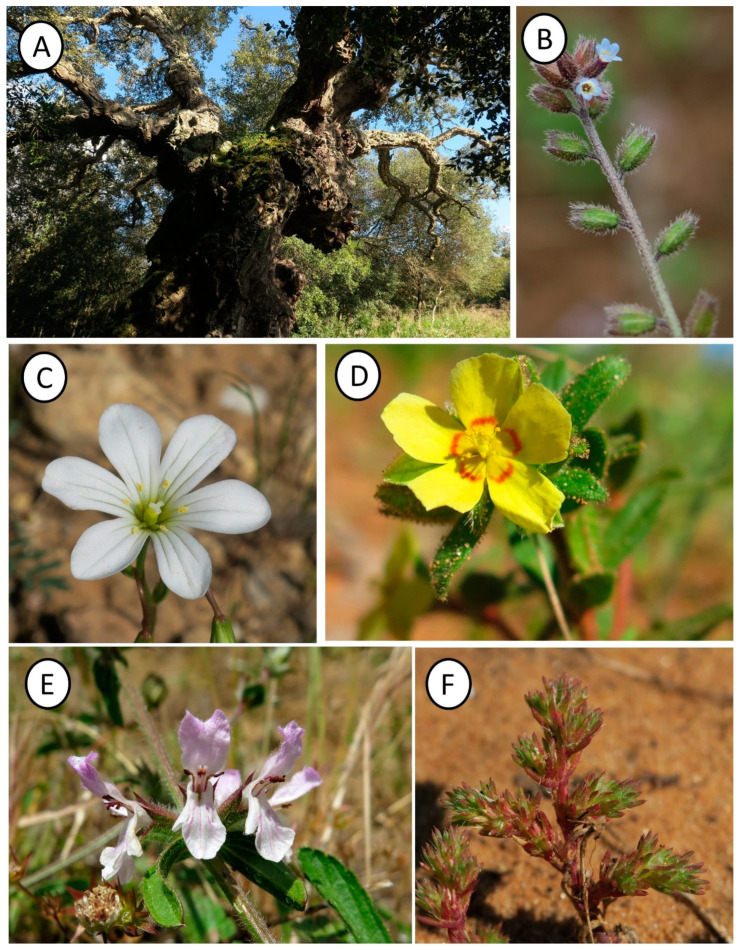
Old *Quercus suber* tree in the Santo Pietro woodland (**A**); *Myosotis congesta* (**B**); *Gagea trinervia* (**C**); *Helianthemum sanguineum* (**D**); *Stachys arenaria* (**E**); *Loeflingia hispanica* (**F**).

**Figure 2:**
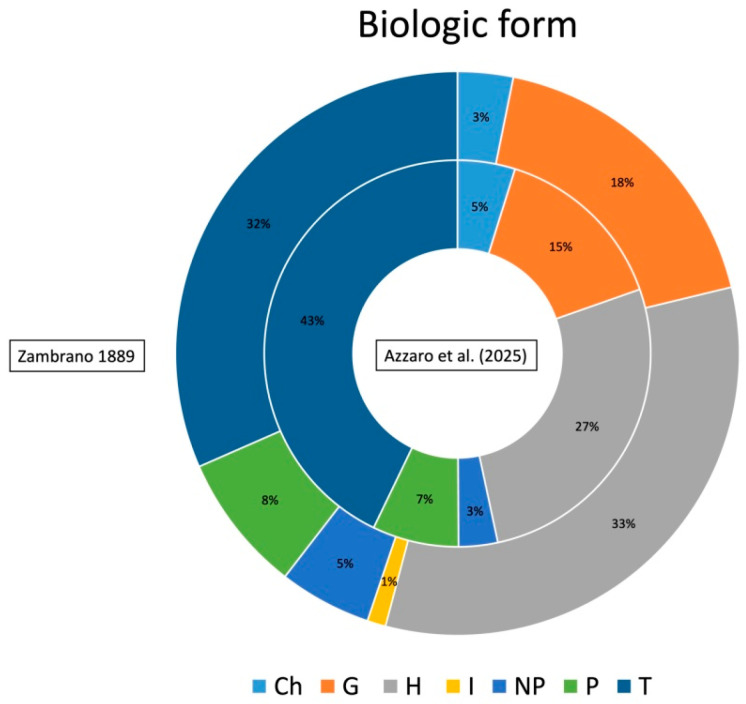
Life form spectrum of the flora of the Santo Pietro woodland according to our results (internal circumference) and Zambrano’s [20] (external circumference).

**Figure 3:**
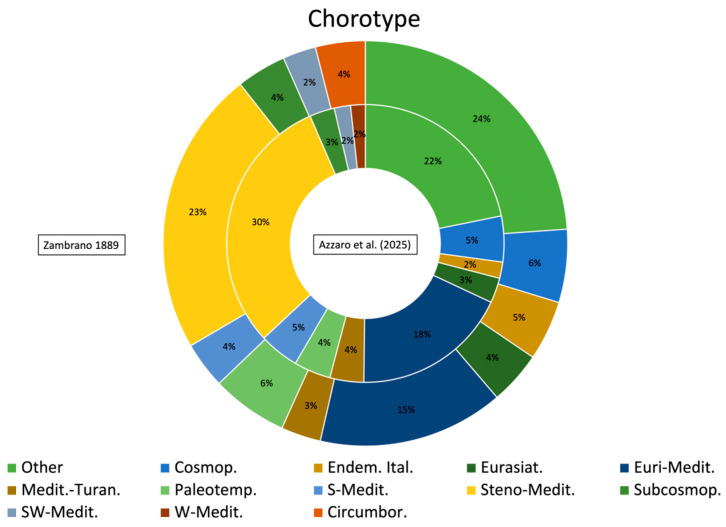
Chorological spectrum of the flora of the Santo Pietro woodland according to our results (internal circumference) and Zambrano’s [20] (external circumference).

**Figure 4:**
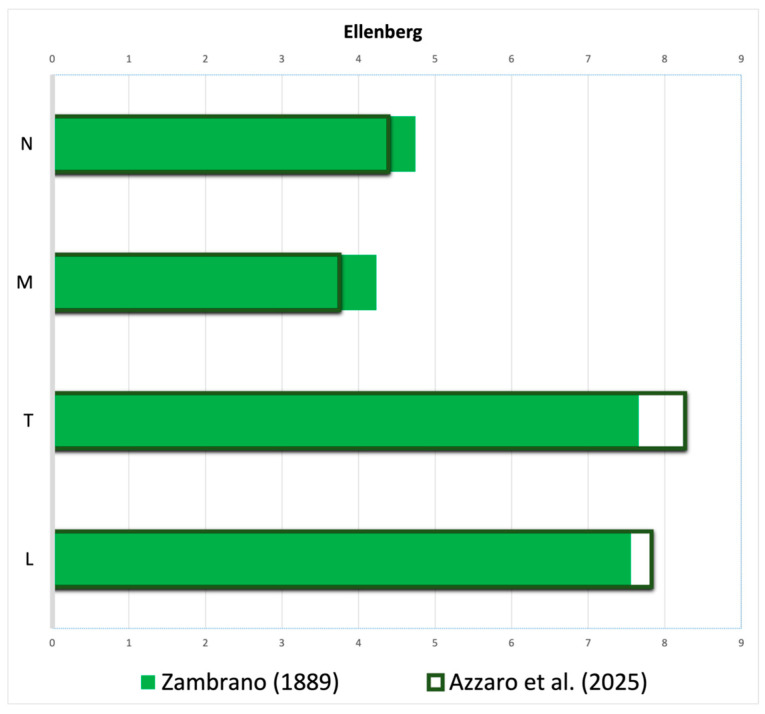
Medium Ellenberg values for the current checklist and the Zambrano list [20]. L = light, T = temperature, M = moisture, N = nutrient.

The floristic checklist of the Bosco di Santo Pietro includes a total of 583 taxa, belonging to 78 families and 339 genera (Table S1, Figure 1). The most represented families are Poaceae (81 taxa), Asteraceae (81 taxa), Fabaceae (42 taxa), Orchidaceae (28 taxa), Lamiaceae (21 taxa) and Asparagaceae (20 taxa), which together make up 46.9% of the total flora. The biological spectrum is dominated by therophytes (42.8%), followed by hemicryptophytes (27%) and geophytes (14.8%). This distribution reflects the adaptation of flora to Mediterranean climatic conditions. The chorological analysis reveals a predominance of Mediterranean elements (69.1%), of which a significant portion are Steno-Mediterranean and Euri-Mediterranean, at 26.6% and 18.2%, respectively. Italian and Sicilian endemism correspond to 1.9% with 11 taxa and 1% with 6 taxa. The Italian endemism is represented by *Aristolochia clusii*, *Biscutella maritima*, *Carduus corymbosus*, *Euphorbia ceratocarpa*, *Gypsophila arrostoi* subsp. *arrostoi*, *Ophrys bertolonii* subsp. *explanata*, *O. exaltata* subsp. *exaltata*, *O. oxyrrhynchos* subsp. *oxyrrhynchos*, *O. sphegodes* subsp. *panormitana*, *O. tenthredinifera* subsp. *grandiflora* and *Ranunculus pratensis*. As regards the Sicilian endemics, *Astragalus caprinus* subsp. *huetii*, *Echium italicum* subsp. *siculum*, *Linaria multicaulis* subsp. *humilis*, *Ophrys subfusca* subsp. *archimedea*, *O. lunulata* and *Senecio glaucus* subsp. *hyblaeus* have been detected. In addition, 52 species are included in the regional or national red lists, of which *Helianthemum sanguineum*, *Myosotis congesta* and *Stachys arenaria* are “Critically Endangered”, *Ephedra fragilis*, *Gagea apulica*, *Juno planifolia*, *Linaria multicaulis* subsp. *humilis* and *Salsola oppositifolia* are classified as “Endangered”, *Carlina involucrata* and *Ophrys bertolonii* subsp. *explanata* are “Vulnerable”, *Ambrosinia bassii*, *Biscutella maritima*, *Chamaerops humilis*, *Squilla pancration*, *Crassula alata*, *Cutandia divaricata*, *Gagea trinervia*, *Helianthemum lippii*, *Neotinea lactea* and *Thymbra capitata* are “Near Threatened” and other 32 species are classified as “Least Concern” [21,22]. In the checklist of vascular plants of the Santo Pietro Forest, 39 alien species have been identified, representing 6.7% of the total species on the list. Among these, 18 taxa are classified as invasive species according to Galasso et al. [23]. As regards the regional rarity, our results show the occurrence of 39 rare or very rare species. Among them, the following species should be mentioned: *Aira multiculmis*, *Callitriche brutia*, *Coris monspeliensis*, *Echinophora tenuifolia*, *Gagea apulica*, *G. lojaconoi*, *G. trinervia*, *Helianthemum aegyptiacum*, *H. lippii*, *H. sanguineum*, *Loeflingia hispanica*, *Myosotis congesta*, *Neatostema apulum*, *Ophrys forestieri*, *O. panormitana*, *Ornithogalum collinum*, *Quercus calliprinos*, *Stachys arenaria* and *Trifolium infamia-ponertii*. The diachronic analysis reveals the disappearance of 178 taxa and the persistence of 199 taxa in the new check list, representing 47% and 53% of 377 taxa listed in the first inventory, respectively. As concerns the life forms (Figure 2), therophytes are slightly less abundant than hemicryptophytes (32% and 33%, respectively), while in our list hemicryptophytes are decidedly less numerous than therophytes (27% and 43%, respectively). Furthermore, in Zambrano’s list, there are four hydrophytes, and in the most recent one, there is only one. As regards the other biological forms, such as geophytes or phanerophytes, no significant differences are noted. The chorological spectrum (Figure 3) reveals some interesting differences: Euromediterranean and Stenomediterranean are dominant in both the lists but with a less significant gap between them in the Zambrano list. In addition, the difference in the number of alien species is very marked, as the Zambrano flora lists only 7 taxa against the 30 listed in our checklist. As concerns the endemic elements, the old list shows a significantly higher number of Italian endemics, while the number of Sicilian endemics remains unchanged. Among the Italian endemics reported by Zambrano and not confirmed by us, the following can be mentioned: *Crocus biflorus*, *Gelasia villosa* subsp. *columnae*, *Jacobaea lycopifolia*, *Myosotis sylvatica* subsp. *elongata*, *Oncostema siculum*, Thalictrum calabricum and Tolpis virgata subsp. grandiflora. The analysis of Ellenberg-type indicator values for the two florae reveals lower medium values of L and T and higher medium values of M and N in the Zambrano flora (Figure 4). In particular, the average value of the L index for our flora is 7.82, while that of the older list is 7.55. As concerns the T index, also in this case a particularly significant increase is noted in the most recent list, going from 7.66 in Zambrano’s list to 8.26 in the current one. The M index instead shows a decrease in the most recent flora (3.75 vs. 4.23). Finally, the N index shows a slight reduction in the current flora, with the change from 4.74 in Zambrano’s list to 4.39 in our list.

## 3. Materials and Methods

### 3.1. Study Area

**Figure 5:**
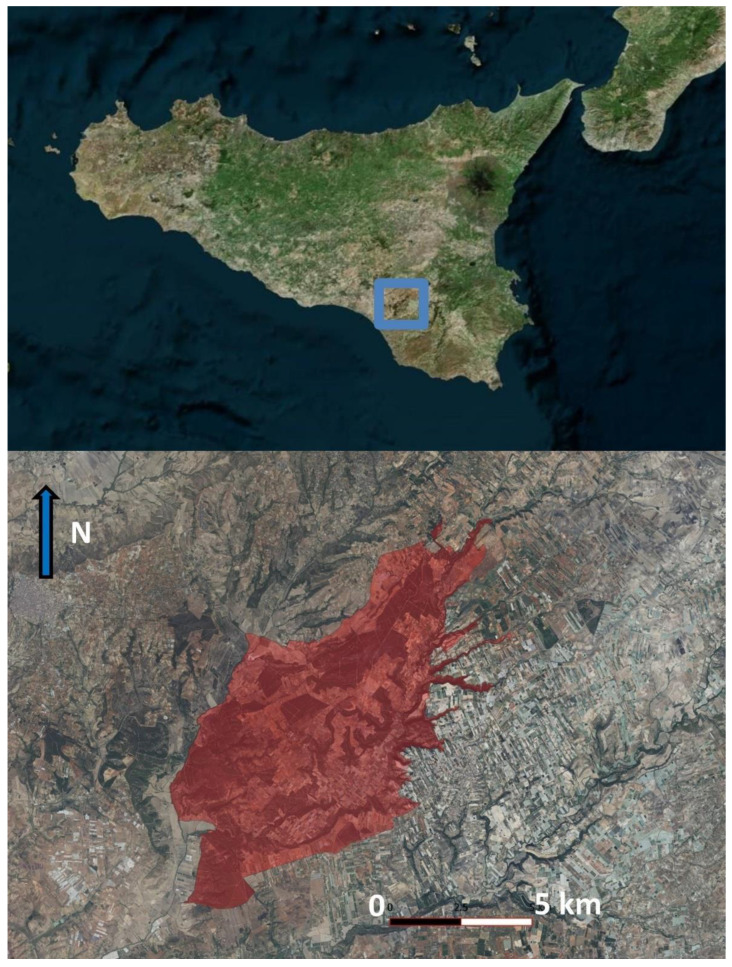
Map of surveyed area with the boundaries of the SAC “Bosco di Santo Pietro” (ITA070005).

The woodland of Santo Pietro is located in the municipality of Caltagirone, in south-eastern Sicily (37°07′ N, 14°30′ E) (Figure 5) and has been known since ancient times: in the Norman era, a vast territory that also included the forest was donated to the people of Caltagirone by King Ruggero; therefore, it has always represented an asset of the city of Caltagirone. Unfortunately, as early as at the end of the 19th century, its decline and poor management were denounced with respect to the rational use of its resources (cork, charcoal, wood, etc.) and a reduction in wooded areas [20]. At the beginning of the 20th century, its partial dismemberment to give plots to farmers accelerated its decline as predicted by Molè [24]. In the following years, reforestation interventions were carried out not aimed at reconstituting and expanding the original forest [25], but at planting exotic species, such as *Eucalyptus* sp. pl, which have partly distorted its characteristics and physiognomy. Regarding its protection in recent times since 1999, the area has been designated as a “nature reserve”, managed by the Regional Forests Company of the Sicilian Region, with the aim of conserving and enhancing this important Mediterranean ecosystem. Unfortunately, in 2007, due to an appeal to the Administrative Justice Council, the establishment decree was annulled, and therefore, the reserve is no longer in force today. However, this area is protected as a Natura 2000 site according to the European Directive 92/43EEC. In fact, the limits of the investigated area coincide with those of the Special Area of Conservation (SAC) “Bosco di Santo Pietro” (ITA070005). This territory covers an area of 7236 hectares, of which 4204 hectares were included in the regional nature reserve “Riserva naturale orientata Bosco di Santo Pietro” and 2079 were classified as pre-reserve [26]. Geomorphologically, the territory is characterized by a sandy plateau with an altitude between 250 and 400 m above sea level, crossed by the valley of the Ficuzza river. The conformation of the land has a typical “a cuestas” morphology, with systems of monoclinal esplanades interrupted by valley incisions [27]. The geological substrate consists mainly of Pleistocene formations, with calcareous and siliceous sands of variable color, clayey intercalations and ancient alluvial deposits [28]. The climate is typically Mediterranean, characterized by hot, dry summers and mild winters. According to the classification of Rivas-Martínez et al. [29], the area falls within the Mediterranean pluviseasonal oceanic bioclimate, with superior thermo-Mediterranean thermotype and superior dry ombrotype. The vegetation is dominated mainly by sparse woodland dominated by *Quercus suber* L., while denser communities with *Quercus ilex* L. cover smaller extensions, restricted to cooler and humid valleys near streams and springs. There are also areas of reforestation with allochthonous species such as eucalyptus and pines as result of past anthropogenic interventions [27]. Some other very remarkable vegetation aspects are represented by thermophilous garrigues and therophytic communities linked to sandy soils. In particular, garrigues are generally interpretable as the result of the degradation of the original vegetation and are represented by communities belonging to *Cisto cretici-Micromerietea julianae* Oberdorfer ex Horvatić 1958 class, which are characterized by the occurrence of *Cistus* sp.pl., *Salvia rosmarinus* Spenn., *Lavandula stoechas* L., *Thymbra capitata* (L.) Cav., *Teucrium fruticans* L., etc. [12,30]. One of the most characteristic and floristically rich types of garrigue in the area is represented by the *Coridothymo capitati-Helichrysetum barrelieri* Barbagallo 1983, where some very rare species such as *Stachys arenaria* and *Gagea trinervia* are present. The annual vegetation of the *Malcolmietalia* Rivas Goday 1958 presents a particular richness of psammophilous species that are rare and often absent in other areas of Sicily [14]. Finally, a very rare and peculiar habitat is represented by the ephemeral pond communities of *Isoëto-Nanojuncetea* Br.-Bl. & Tüxen ex Westhoff, Dijk & Passchier 1946 class [31].

### 3.2. Methods

The elaboration of the floristic checklist of the Santo Pietro woodland was conducted through an integrated approach that combined field research, analysis of historical herbariums and a review of the existing literature. Plant specimens were deposited at the Herbarium of the University of Catania (CAT).

At the same time, the historical herbariums relevant to the area were consulted, such as *Herbarium* of the University of Catania (CAT) and *Herbarium Mediterraneum Panormitanum* (PAL). This made it possible to compare our data with historical reports of species not detected during field research. A systematic review of the floristic literature concerning the study area was also conducted, including both scientific publications and unpublished technical reports.

The nomenclature of the species has been updated according to the checklists of the native Italian vascular flora [32], except for the genus *Quercus* for which we follow Pignatti [33], and Italian alien species [23]. For each taxon, information on biological form, chorotype and conservation status according to IUCN categories are reported in Table S1. Conservation data follow Orsenigo et al. [21] and Rossi et al. [22]. In addition, the regional rarity of each taxon, according to the classification system of plant species in Sicily proposed by Giardina et al. [34], is reported.

The categories are as follows: EX: Extinct; RR: Very Rare; R: Rare; NC: Uncommon; C: Common; CC: Very Common.

Ellenberg-type indicator values (light, temperature, moisture and nutrient) were obtained from values reported for Italy [35], also updated in Tichý et al. [36].

## 4. Discussion and Conclusions

### 4.1. Checklist of Santo Pietro Flora

The results obtained highlight the remarkable floristic richness of the Santo Pietro woodland, confirming its role as a local hotspot in Sicily. The number of taxa detected (583) is significantly higher than that reported in previous studies [12,20], demonstrating the importance of an integrated and prolonged research approach.

The predominance of therophytes in the biological spectrum is in line with the expected result for Mediterranean ecosystems [37], reflecting the adaptation of flora to summer aridity conditions. The significant presence of species included in the red lists (8.9%) underlines the conservation value of the area, which is configured as an important refuge for species more at risk than a significant part of the flora of the Santo Pietro woodland, suggesting the need to implement targeted conservation measures [38].

The significant number of endemic and rare species also highlights the potential of this area as a refuge for threatened species and underlines the importance of in-depth floristic studies even in apparently well-known areas [39].

On the other hand, alien naturalized species represent a significant component of the flora of the Santo Pietro woodland. As emphasized by several authors [40,41,42], a trend of increased invasiveness was observed in Sicily and nearby islets. Our data show an incidence of alien species of up to 6.7% of the total flora of the area, with 39 taxa identified. This figure is in line with the Sicilian regional average [34] and reflects the increasing pressure of biological invasions on Mediterranean ecosystems [43].

Among the alien species detected, *Acacia saligna*, (Labill.) H.L.Wendl *Ailanthus altissima* (Mill.) Swingle and *Eucalyptus camaldulensis* Dehnh. deserve particular attention [44]. These species, introduced mainly for reforestation and erosion control purposes, have shown a significant ability to spread, altering the composition and structure of native plant communities or habitats [45,46,47].

The presence of alien species raises important conservation issues. On the one hand, some of these species can provide ecosystem services, such as carbon sequestration or soil stabilization. On the other hand, they can compete with native species, alter biogeochemical cycles and modify disturbance regimes, particularly those related to fires [45]. Continuous monitoring of the presence and spread of alien species is essential for the management of the protected area. Control and, where possible, eradication strategies for the most invasive species should be implemented, paying particular attention to areas of greater conservation value [48]. At the same time, it is necessary to prevent new introductions through awareness and regulation programs of human activities in the area.

However, the relatively low percentage of alien species (6.7%) compared to other similar Mediterranean areas [49] suggests that the forest of Santo Pietro still maintains a good degree of ecological integrity. However, the presence of highly invasive species (18 different taxa) requires constant attention and the implementation of adaptive management strategies to preserve the native biodiversity of this important Mediterranean ecosystem.

In conclusion, this checklist provides a fundamental knowledge base for future ecological and biogeographical research in the area. The results obtained support the need to maintain and strengthen the protection measures currently in force, in order to preserve this important heritage of Mediterranean plant biodiversity [50].

### 4.2. Diachronic Analysis

Our comparison with the list reported by Zambrano [20] allows for some interesting observations. First of all, a notable difference in the number of taxa detected is highlighted: 377 in Zambrano and 583 in the present checklist. This difference can probably be explained by the limited floristic knowledge at the time and by more in-depth field investigations. The sampling intensity (frequency of visits and surface explored) allows us to significantly increase the number of taxa recorded as, for comparison, happened in the Maddalena peninsula near Syracuse where, approximately 30 years after the first floristic census, there was an increase of almost 200 species with a doubling of the overall list [51]. However, in the case of Santo Pietro, also considering the long period of time (135 years) that elapsed between the two floristic studies, the two lists show significant differences not only from a quantitative, but also qualitative, point of view attributable to changes in environmental conditions. In particular, it can be noted that the loss of several hygrophilous and nemoral species is probably due to climate change and habitat degradation. In fact, Zambrano reported the presence of some mesophilous and/or hygrophilous species, such as *Osmunda regalis* L., *Myosurus minimus* L., *Aquilegia vulgaris* L., *Pseudoturritis turrita* L., *Potamogeton fluitans* Roth. (=*P. nodosus* Poir.), *P. densus* L. (=*Groenlandia densa* (L.) Fourr.), *Zannichellia palustris* L., *Sparganium ramosum* Huds. (=*S. erectum* L.?), *Scolopendrium officinale* Suar. (=*Asplenium scolopendrium* L.) and *Cephalanthera pallens* Rich. (=*C. damasonium* (Mill.) Druce). Others have reported with great interest *Ionopsidium albiflorum* Dur. and *Pteris cretica* L., very rare species in Sicily that have not been recently found on the island [34]. The reporting of these taxa is particularly significant as they are evidence of permanent humid environments that have now disappeared and there is no other information in the literature. In addition, these records attest the past presence of nemoral mesophilous species, which currently in Sicily survive only in mountain areas, mainly in the northern part of the island.

At the same time, the increase in alien species in our checklist suggests an increase in anthropic pressure in various forms, especially due to reforestation, degradation of natural environments, fires that have favored the spread of numerous woody invasive plants, such as *Acacia saligna*, *Ailanthus altissima*, *Vachellia karroo*, etc.

The results obtained from the comparison between the mean values of the Ellenberg indices of the two floras highlight the significant ecological modifications occurring in the last century in this area.

In particular, a lower average value of L can be seen in Zambrano’s list. This can be easily explained by considering the current disappearance of many nemoral species with a low light requirement that used to find their habitat in closed and dense forest formations now largely replaced by sparse and open woodland or scrubs. The increase in thermophilous species and the rarefaction of the more mesophilic ones can be related both to the degradation of forest environments and to a general global tendency linked to climate change. Other species could still disappear in the coming years if the trend towards the climate drying in Sicily were to remain or worsen [52,53,54]. The decrease in M index in our list is almost certainly correlated to the disappearance of numerous hygrophilous species and the habitats associated with them. In fact, currently, the only natural humid environments represented in the area are small temporary ponds of the *Isoeto-Nanojuncetea* class, while the occurrence of certain species reported by Zambrano such as *Osmunda regalis*, *Zannichellia palustris*, *Sparganium ramosum*, etc., suggest the ancient presence of wetlands with permanent water having disappeared. The slight reduction in the N index observed in our list may perhaps be related to the significant abandonment of agricultural areas that has favored the replacement of cultivated areas in favor of grasslands, garrigues and shrublands. In trying to make a comparison with other diachronic studies on the changes in flora, many of these have been conducted on the high mountains of Europe, especially on the Alps [55,56,57], where climatic changes seem more evident and the plants are arranged on an altitudinal gradient, so they are subject to an upward shift in their distribution. However, these shifts due to climatic warming have opposite effects on the summit floras’ species richness: increasing in boreal–temperate mountain regions and decreasing in Mediterranean mountain regions, probably because recent climatic trends have decreased the availability of water in the European Mediterranean mountains [58]. In general, on the Alps and high Mediterranean mountains, human action seems less relevant than climatic warming [59], while in the Mediterranean lowlands and smaller islands, recent climate changes overlap with intense and direct human actions of various intensity sustained over time [60]. Many diachronic studies of flora have concentrated on small Mediterranean islands [61,62,63,64], places on which a vast collection of literature dating back to the last two centuries is available, allowing comparisons to be made, while diachronic studies on the flora of continental hilly and lowland Mediterranean areas or larger islands are few [65,66]. These long-term studies provide a valuable framework for identifying broad patterns of flora composition change over time and predicting the impacts of climate change on the biodiversity of mediterranean environments, where more research is, however, needed.

## Data Availability

The original contributions presented in this study are included in this article/Supplementary Materials. Further inquiries can be directed to the corresponding authors.

## Supplementary Materials

The following supporting information can be downloaded at https://www.mdpi.com/article/10.3390/plants14050788/s1, Table S1: Checklist of vascular flora of the Santo Pietro woodland.
