# Supplementary material for: Diachronic Analysis of the Floristic Diversity of the Special Area of Conservation (SAC) “Bosco di Santo Pietro” (South-Eastern Sicily): A Mediterranean Biodiversity Hotspot
Source: Plants (Basel). 2025 Mar 4;14(5):788. doi: 10.3390/plants14050788 (PMC11901573; doi:10.3390/plants14050788)
Supplement: Supplementary file 1 [file plants-14-00788-s001.zip › plants-3486281-supplementary.pdf]

plants
PLANTS 14, 2025

Diachronic analysis of the floristic diversity of the Special Area of Conservation (SAC) "Bosco di Santo Pietro" (South-eastern Sicily): a Mediterranean Biodiversity Hotspot

Dario Azzaro, Salvatore Cambria, Manuela Porrovecchio and Pietro Minissale
Department of Biological, Geological and Environmental Sciences, University of Catania, Italy

Table S1: Vascular Flora Checklist of the Santo Pietro Woodland

Species	Biological form	Chorotype	Red List	Aliens	Azzaro's Flora (A)	Zambrano's Flora (Z)	Note	L	T	M	N
Acacia saligna (Labill.) H.L. Wendl.	P	Australia		X	A			8,5	9	5	4
Acanthus mollis L.	H	Steno-Medit.-Occid.			A			6	8,3	3,5	7,5
Achillea ligustica L.	H	Steno-Medit.-Occid.				Z		7,5	9	4	4,5
Achillea millefolium L.	H	Eurosib.				Z	in Sicilia non risulta presente	7,5	9	4	4,5
Achnatherum bromoides (L.) P. Beauv.	H	Steno-Medit.			A			7,8	9	3,5	2
Acis autumnalis (L.) Sweet	G	Steno-Medit.				Z	sub Leucojum autumnale L.	9	9	1	2
Adiantum capillus-veneris L.	G	Subtrop.			A	Z		3	8	7,6	2,8
Adonis annua L. subsp. *cupaniana* (Guss.) Steinb.	T	Euri-Medit.				Z	*Adonis cupaniana* Guss.	7,6	7	4	5
Aegonychon purpurocaeruleum (L.) Holub	H	S-Europ.				Z	sub *Lithospermum purpurocaeruleum* L.	5,4	7	3,9	3,9
Agave americana L.	P	N-Americ.		X	A	Z		8,5	10	3	5,5
Agave salmiana subsp. *ferox* (K. Koch) Hochstätter	P	C-Americ.		X (N)	A			8,5	10	3	5,5
Agrimonia eupatoria L.	H	Eurasiat.			A			7,1	6	4,1	3,9
Agrostis castellana Boiss. & Reut.	H	Euri-Medit.-Occid.			A			8,3	7,1	4	2,7
Ailanthus altissima (Mill.) Swingle	P	Asiatica		X (I)	A			7,1	7	5,1	7,2
Aira cupaniana Guss.	T	Steno-Medit.-Occid.			A			7,5	9	2	1
Aira multiculmis Dumort.	T	Subtrop.		X	A			8,7	6,5	2,4	1,6

Ajuga chamaepitys (L.) Schreb.	H	Steno-Medit.			A			7,5	8	3,6	3
Ajuga iva (L.) Schreb.	Ch	Steno-Medit.			A			8	8	3,3	4,3
Ajuga orientalis L.	H	E-Medit.			A			6,8	8	4,2	6
Alcea rosea L.	H	ignota			A			8	7,1	4,3	6,5
Alkanna tinctoria Tausch	H	Steno-Medit.			A			8	9,7	1,7	2
Allium chamaemoly L. subsp. *chamaemoly*	G	Steno-Medit.-Sudoccid.	LC		A			7,5	9	3	3
Allium neapolitanum Cirillo	G	Steno-Medit.				Z		7,3	9,3	4,3	6,7
Allium nigrum L.	G	Steno-Medit.			A			8	9,3	4	6,3
Allium polyanthum Schult. & Schult. f.	G	Avv.			A			7	7,7	4	5
Allium roseum L. subsp. *roseum*	G	Steno-Medit.	LC		A	Z		7,4	8,6	3,4	5
Allium sardoum Moris	G	Steno-Medit.			A			7,8	8	3,6	2,5
Allium subhirsutum L.	G	Steno-Medit.-Occid.			A			7	9,3	4	6,3
Amaranthus albus L.	T	N-Americ.		X (I)	A	Z		8,4	9	3,7	7,3
Amaranthus blitum L.	T	Cosmop.				Z		7,9	8	4,4	7,9
Amaranthus graecizans L.	T	Paleotemp.		X (I)	A			8,5	8	4,1	7,9
Amaranthus retroflexus L.	T	Cosmop.		X (I)	A			8	9	4,6	7,9
Amaranthus viridis L.	T	S-Americ.		X (I)	A			8	9	4,7	8
Ambrosinia bassii L.	G	Steno-Medit.-Occid.	NT		A			7	7,5	4	4,5
Ammoides pusilla (Brot.) Breistr.	T	Steno-Medit.			A			7	9	2	2
Ampelodesmos mauritanicus (Poir.) T. Durand & Schinz	H	Steno-Medit.-Sudoccid.	LC		A			8,5	10	2	3
Anacamptis collina (Banks & Sol. ex Russell) R.M. Bateman, Pridgeon & M.W. Chase	G	Steno-Medit.			A	Z	sub Orchis saccata Ten.	8	9	4	3,3
Anacamptis longicornu (Poir.) R.M. Bateman, Pridgeon & M.W. Chase	G	Steno-Medit.-Occid.			A	Z	sub Orchis longicornu Poir.	7,6	6,5	3	3,1
Anacamptis papilionacea (L.) R.M. Bateman, Pridgeon & M.W. Chase	G	Euri-Medit.			A	Z	sub Orchis papilionacea L.	7,7	8	3,7	4
Anacyclus clavatus (Desf.) Pers.	T	Steno-Medit.			A			8,3	8	2,7	5,7
Anagyris foetida L.	P	S-Medit.				Z		7,7	8	3,3	5,3
Anchusa azurea Mill.	H	Euri-Medit.			A	Z	sub Anchusa azurea Retz.	8,7	8	3,5	5,2
Andropogon distachyos L.	H	Paleotrop.			A	Z		8,7	9	2,7	3,3

Anemone apennina L.	G	S-Europ.				Z		4,5	5,5	5	5
Anemone coronaria L.	G	Steno-Medit.				Z		7,3	8,3	4,7	4,3
Anemone hortensis L.	G	Euri-Medit.-Sett.			A			7,3	8,3	4,3	3,7
Anisantha diandra (Roth) Tutin ex Tzvelev	T	Euri-Medit.			A	Z	sub Bromus gussonei Parl.	7,8	8	3,2	6
Anisantha fasciculata (C. Presl) Nevski	T	S-Medit.			A			8,5	11	2	2
Anisantha madritensis (L.) Nevski	T	Euri-Medit.			A			7,8	7	2,8	6
Anisantha rigida (Roth) Hyl.	T	Paleosubtrop.			A			8,2	6	3	5
Anisantha rubens (L.) Nevski	T	Medit.-Turan.			A			8,3	11	2	5
Anisantha sterilis (L.) Nevski	T	Medit.-Turan.			A	Z	sub Bromus sterilis L.	7,3	7	4	5
Anisantha tectorum (L.) Nevski	T	Paleotemp.			A			8,2	6	3	4
Anthemis arvensis subsp. *incrassata* (Loisel.) Nyman	T	Steno-Medit.			A			7,4	6	4	6
Anthemis cotula L.	T	Eurasiat.				Z		7,3	7	4	5
Anthemis cretica L. subsp. *columnae* (Ten.) Franzén	H	Medit.-Mont.				Z	sub Anthemis montana L.	8,5	4	3	1,6
Anthoxanthum odoratum L.	H	Eurasiat.			A			7,3	3,9	5	3,2
Anthyllis vulneraria subsp. *rubriflora* (DC.) Arcang.	H	Euri-Medit.			A			7,9	5	3,8	2,6
Antirrhinum siculum Mill.	Ch	Endem. Ital.				Z		9	10	2	1
Aphanes arvensis L.	T	Subcosmop.			A			6,8	5	6	4,9
Aquilegia vulgaris L.	H	Eurasiat.				Z	segnalazione dubbia	6,3	5,9	4,8	4,6
Arbutus unedo L.	P	Steno-Medit.			A			7,3	9	3,6	2
Arenaria leptoclados (Rchb.) Guss.	T	Cosmop.			A			8,7	9	2,5	2,6
Arenaria serpyllifolia L.	T	Cosmop.			A			8,3	5	3,5	4
Arisarum vulgare O. Targ.Tozz.	G	Steno-Medit.			A			6	9	4	6,7
Aristolochia clusii Lojac.	G	Endem. Ital.			A	Z	sub Aristolochia pallida non W	8	8	4	4
Aristolochia sempervirens L.	P	SW-Medit.				Z		5	11	5	5
Artemisia arborescens L.	NP	S-Medit.				Z		8,7	10	5	6
Arum italicum Mill.	G	Steno-Medit.			A	Z		6	8	4	6,1
Arundo donax L.	G	Subcosmop.			A			8,3	9,3	6,7	7
Asparagus acutifolius L.	G	Steno-Medit.			A	Z		6,3	8,8	2,3	4
Asparagus albus L.	Ch	Steno-Medit.-Occid.	LC		A	Z		7	9	3	3
Asparagus horridus L.	NP	S-Medit.			A			8,5	11	1,5	2,5

Asphodelus fistulosus L.	H	Subtrop.			A			8,3	9	3	6,3
Asphodelus ramosus L.	G	Steno-Medit.			A	Z		8	8,5	3,3	5,3
Asplenium ceterach L.	H	Eurasiat.			A			7,8	7,5	3,3	5,3
Asplenium scolopendrium L. subsp. *scolopendrium*	H	Circumbor.				Z	sub Scolopendrium officinale L.	2	6	5	6
Asteriscus aquaticus (L.) Less.	T	Steno-Medit.			A			9	10	7	7
Astragalus boeticus L.	T	S-Medit.			A			8,5	11	2,5	3
Astragalus caprinus subsp. *huetii* (Bunge) Podlech	H	Endem. Sic.	LC		A			9	10	3	2
Astragalus hamosus L.	T	Medit.-Turan.			A			7,8	10	3,8	6
Astragalus sesameus L.	T	Steno-Medit.				Z		9	9	2	2
Atractylis cancellata L.	T	S-Medit.			A	Z		8,5	9,5	2	3,5
Atriplex halimus L.	P	Steno-Medit.				Z		8,3	10	6	6,3
Atriplex patula L. subsp. *patula*	T	Circumbor.				Z		6,9	5,8	5	8
Austrocylindropuntia subulata (Muehlenpf.) Backeb.	P	S-Americ.		X (N)	A			9	9	2	6
Avellinia festucoides (Link) Valdés & H. Scholz	T	Steno-Medit.			A			8,5	10	2	3,5
Avena barbata Pott ex Link	T	Medit.-Turan.			A	Z		8	8,5	2,9	2
Avena fatua L.	T	Eurasiat.			A			7,3	10	4,5	6
Avena sterilis L.	T	Medit.-Turan.			A	Z		8	9	3,3	5,5
Ballota nigra subsp. *uncinata* (Bég.) Patzak	H	Euri-Medit.			A	Z	sub Ballota foetida Lam.	7,4	6	4,7	7,5
Barlia robertiana (Loisel.) Greuter	G	Steno-Medit.	LC		A	Z	sub Orchis longebracteata Biv.	7,3	9,3	4	5
Bellardia trixago (L.) All.	T	Euri-Medit.			A			8	8,5	3	3,7
Bellardia viscosa (L.) Fisch. & C.A. Mey.	T	Medit.-<br>Atl.(Euri-)			A			8	8,8	5	5
Bellevalia dubia (Guss.) Rchb.	G	Endem. Ital.				Z	sub Hiacinths dubium L.	7	9	4	4
Bellevalia romana (L.) Sweet	G	Centromedit.				Z	sub Hiacinthus romanus L.	8	7,7	6,5	4,5
Bellis annua L.	T	Steno-Medit.			A			7	9,7	6,5	3,5
Bellis perennis L.	H	Circumbor.			A	Z	incl. Bellis hybrida Ten.	8,1	5	5	5
Bellis sylvestris Cirillo	H	Steno-Medit.			A	Z		6,9	8	3	2,8
Beta vulgaris L.	H	Euri-Medit.			A			8,5	7	5,1	7,7
Biarum tenuifolium (L.) Schott<br>subsp. *abbreviatum* (Schott) K.Richt.	G	Steno-Medit.			A			5,8	9	4	5,8
Biscutella maritima Ten.	T	Endem. Ital.	NT		A	Z	Biscutella lyrata L.	9	10	2	1
Bituminaria bituminosa (L.) C.H. Stirt.	H	Euri-Medit.			A	Z	sub Psolarea bituminosa L.	7,9	9	3,7	4

Blackstonia acuminata (W.D.J. Koch & Ziz) Domin subsp. *acuminata*	T	Medit.			A			8,1	9	7	2
Bolboschoenus maritimus (L.) Palla	G	Cosmop.				Z	sub Scirpus maritimus L.	8	6	9,6	6,2
Borago officinalis L.	T	Euri-Medit.			A	Z		7,8	8	3,6	6,4
Brachypodium distachyon (L.) P. Beauv.	T	Medit.-Turan.			A			8	9	1,5	2,5
Brachypodium sylvaticum (Huds.) P. Beauv.	H	Paleotemp.			A			4	5	5,4	5,6
Brassica nigra (L.) W.D.J.Koch	T	Euri-Medit.				Z	Sinapis nigra L.	8,1	7	6	6,7
Brassica souliei (Batt.) Batt. subsp. *amplexicaulis* (Desf.) Greuter & Burdet	T	SW-Medit.				Z	*Brassica amplexicaulis* (Desf.) Pomel	9	11	1	1
Briza maxima L.	T	Paleosubtrop.			A			7,5	9,3	2	2,3
Briza minor L.	T	Subcosmop.				Z		7,8	9	7	4,5
Bromus alopecuros Poir. subsp. *alopecuros*	T	Steno-Medit.	LC		A			8	9	2	5,5
Bromus hordeaceus L.	T	Subcosmop.			A	Z	sub Bromus mollis L.	7,6	6	1	6
Bunias erucago L.	T	Euri-Medit.-Sett.			A			8,2	8	3,6	6
Bupleurum semicompositum L.	T	Medit.-Turan.			A			8,5	11	4,5	3
Cachrys cristata DC.	H	E-Medit.			A			8,5	10	3,5	6,5
Cachrys libanotis L.	H	NW-Medit.			A			9	9	3	5
Calendula arvensis (Vaill.) L.	H	Euri-Medit.			A	Z	incl. Calendula parviflora Raf.	7,5	8,3	3,3	6
Callitriche brutia Petagna	I	Subatl.			A			8,3	6,8	10	4,5
Campanula dichotoma L.	T	Steno-Medit.-Occid.			A			7	8	4	5
Campanula erinus L.	T	Steno-Medit.			A			8	8,8	2,3	3,8
Capsella bursa-pastoris (L.) Medik.	H	Cosmop.			A			7,4	5,2	4,7	4
Cardamine hirsuta L.	T	Cosmop.			A			6,5	8	3	4
Carduus argyroa Biv.	T	Steno_				Z		7	8	3	7
Carduus cephalanthus Viv.	T	Steno-Medit.				Z	sub Carduus congestus Guss.	8,5	10	4	7
Carduus collinus Waldst. & Kit. subsp. *collinus*	H	SE-Europ.				Z	sub Cnicus pungens Willd. Non presente in Sicilia	7,5	5	4	4
Carduus corymbosus Ten.	T	Endem. Ital.	LC		A	Z		7	8	3	7
Carduus pycnocephalus L. subsp. *pycnocephalus*	H	Medit.-Turan.			A	Z		7,9	8	3	7
Carex caryophyllea Latourr.	H	Eurasiat.			A			7,9	5	4	2
Carex distachya Desf.	H	Steno-Medit.			A	Z	sub Carex linkii Willd.	6,8	6	4	4

Carex divulsa Stokes	H	Euri-Medit.			A	Z		6,5	6,5	3,5	5
Carex flacca subsp. *erythrostachys* (Hoppe) Holub	G	Europ.			A			7,2	5	6	2,8
Carex pendula Huds.	H	Eurasiat.			A	Z	sub Carex maxima Scop.	8	5	8	5,9
Carex riparia Curtis	G	Eurasiat.				Z		6,9	5	10	5,4
Carlina involucrata Poir.	H	S-Medit.	VU		A			9	11	1	1
Carlina lanata L.	T	Steno-Medit.			A	Z		7,5	7	3	5,5
Carthamus caeruleus L.	H	S-Medit.			A			8,5	11	4	4,5
Carthamus lanatus L.	T	Euri-Medit.			A			8,5	8	3	5,7
Catabrosa aquatica (L.) P.Beauv.	H	Circumbor.				Z	sub Glyceria ochroleuca Dumor	8	4	8,8	8
Catapodium rigidum (L.) C.E. Hubb. subsp. *rigidum*	T	Euri-Medit.			A			8,4	8	2	5
Catapodium rigidum subsp. *majus* (C. Presl) F.H. Perring & P.D. Sell	T	Euri-Medit.			A			8,4	8	2	5
Celtis australis L.	P	Euri-Medit.			A			7,5	8	3	4
Centaurea calcitrapa L.	H	Euri-Medit.			A	Z		8,3	9	3	5
Centaurea jacea L. subsp. *gaudinii* (Boiss. & Reut.) Gremli	H	Orof. S-Europ.				Z	sub Centaurea amara L.	7,6	5	5,3	5
Centaurea napifolia L.	T	Steno-Medit.-Sudoccid.			A	Z		8	9,5	3,5	6,5
Centaurea sicula L.	H	SW-Medit.			A	Z	sub Centaurea fuscata Desf.	9	9,5	2	5
Centaurea solstitialis L. subsp. *solstitialis*	H	Steno-Medit.			A	Z	sub Centaurea lappacea Ten.	8,3	6	2	6,2
Centaurium erythraea Rafn	H	Paleotemp.			A			7,9	6	5	5
Centaurium grandiflorum subsp. *majus* (Hoffmanns. & Link) Z. Díaz	H	Steno-Medit.			A			7,9	6	5	5
Centaurium maritimum (L.) Fritsch	T	Steno-Medit.-Occid.			A			8,7	9	5	2
Centranthus ruber (L.) DC.	Ch	Steno-Medit.			A			7,1	8	2	4
Cephalanthera damasonium (Mill.) Druce	G	Euri-Medit.				Z	sub Cephalantera pallens Rich.	2	5	4	4
Cerastium glomeratum Thuill.	T	Cosmop.			A	Z		7	6	5	5
Ceratonia siliqua L.	P	Steno-Medit.			A	Z		9	11	3	4
Cerinthe major L.	T	Steno-Medit.			A	Z	sub cerinthe aspera L.	7	8	4	8
Cerinthe minor L. subsp. *auriculata* (Ten.) Domac	T	Subendem.				Z	sub Cerinthe maculata L.	7	3	4	5
Chamaerops humilis L.	NP	Steno-Medit.-Occid.	NT		A	Z		9	10	3	4
Chelidonium majus L.	H	Circumbor.				Z		6	6	5	8

Chenopodium album L.	T	Cosmop.			A	Z		7	7	4	7
Chenopodium opulifolium Schrad. ex W.D.J. Koch & Ziz	T	Paleotemp.			A			8	7	3	6
Chenopodium vulvaria L.	T	Euri-Medit.			A	Z		7	7	4	9
Chondrilla juncea L.	H	Euri-Medit.				Z		8	7	3	5
Chrozophora tinctoria (L.) A. Juss.	T	Medit.-Turan.			A			8	12	2	7
Cichorium endivia L. subsp. *pumilum* (Jacq.) Cout.	T	Steno-Medit.				Z	sub Cichorium divaricatum Schousb.	9	9	3	4
Cichorium intybus L.	H	Cosmop.			A			9	6	3	5
Cirsium arvense (L.) Scop.	G	Eurasiat.				Z	sub Cnicus arvensis Sm.	8	5	4	7
Cirsium creticum subsp. *triumfettii* (Lacaita) K. Werner	H	Orof. NE-Medit.			A			7	6	7	4
Cirsium vulgare (Savi) Ten.	H	Eurasiat.				Z	sub Cnicus lanceolatus (L.) Willd.	8	5	5	8
Cistus creticus L.	NP	Centromedit.			A	Z		9	9	2	2
Cistus creticus L. subsp. *eriocephalus* (Viv.) Greuter & Burdet	NP	Steno-Medit.				Z	Cistus villosus L.	9	9	2	2
Cistus crispus L.	NP	W-Medit.				Z	erroneous record				
Cistus monspeliensis L.	NP	Steno-Medit.			A	Z		9	9	2	2
Cistus salviifolius L.	NP	Steno-Medit.			A	Z		9	11	2	3
Cladanthus mixtus (L.) Chevall.	T	Steno-Medit.			A			8	8	2	1
Clematis cirrhosa L.	P	Medit.-Turan.				Z		7	10	4	5
Clematis vitalba L.	P	Europ.			A	Z		7	7	5	7
Clinopodium nepeta (L.) Kuntze subsp. *nepeta*	Ch	Medit.-Mont.			A	Z	sub Thymus nepeta Sm.	5	7	3	6
Clinopodium vulgare subsp. *arundanum* (Boiss.) Nyman	H	Circumbor.			A			7	5	4	3
Colchicum cupanii Guss. subsp. *cupanii*	G	Steno-Medit.	LC		A			8	11	3	4
Coleostephus myconis (L.) Cass. ex Rchb. f.	T	Steno-Medit.			A			8	9	4	4
Conium maculatum L.	H	Paleotemp.				Z		7	8	4	7
Convolvulus althaeoides L.	H	Steno-Medit.-Occid.			A			8	9,5	3	7
Convolvulus arvensis L.	G	Cosmop.			A	Z		7	7	4	5
Convolvulus cantabrica L.	H	Euri-Medit.			A			9	8	3	2
Convolvulus sepium L.	H	Eurasiat.				Z	sub Calystegia sepium (L.) R. Br prob . Confusa con la precedente	7	8	7	7
Convolvulus silvaticus Kit.	H	SE-Europ.			A	Z	sub Calystegia sylvatica (Kit.) Griseb.	7	8	7	7

Coris monspeliensis L.	H	Steno-Medit.-Occid.			A			9	9	2	2
Coronilla repanda (Poir.) Guss.	T	W-Medit.			A	Z		9	9	2	2
Coronilla scorpioides (L.) W.D.J. Koch	T	Euri-Medit.			A			9	9	2	3
Coronilla valentina L.	NP	SW-Medit.			A			9	11	2	3
Corynephorus articulatus (Desf.) P. Beauv.	T	Steno-Medit.			A			8	9	2	1
Crassula alata (Viv.) A. Berger	T	Criptogenica	NT		A			8	6	6	3
Crataegus monogyna Jacq.	P	Eurasiat.			A	Z	sub Crataegus oxyacantha L.	6	7	4	5
Crepis bursifolia L.	H	Steno-Medit.				Z		9	6	3	7
Crepis foetida L. subsp. *foetida*	T	Euri-Medit.			A			9	9	5	5
Crepis leontodontoides All.	H	W-Medit.			A			7	8	4	7
Crepis vesicaria L.	H	Subatl.			A	Z		8	8	3	5
Crocus biflorus Mill.	G	Endem. Ital.				Z		8	6	4	4
Crocus longiflorus Raf.	G	Subendem.	LC		A	Z		9	8	2	2
Crupina crupinastrum (Moris) Vis.	T	Steno-Medit.			A			9	8	2	2
Cutandia divaricata (Desf.) Barbey	T	Steno-Medit.-Sudoccid.	NT		A			9	11	1	3
Cyclamen hederifolium Aiton	G	Steno-Medit.-Sett.			A			4	8	5	5
Cyclamen repandum Sm.	G	N-Medit.			A			4	9	3	5
Cymbalaria muralis G.Gaertn., B.Mey. & Scherb. subsp. *muralis*	Ch	Subcosmop.				Z	sub *Linaria cymbalaria Mill.*	8	7,5	3	2,5
Cynanchica aristata (L.f.) P.Caputo & Del Guacchio subsp. *scabra* (Nyman) P.Caputo & Del Guacchio	H	Steno-Medit.				Z	sub Asperula longiflora Wald. & Kit.	9	8	2	2
Cynara cardunculus L. subsp. *cardunculus*	H	Steno-Medit.			A	Z	sub Cynara scolymus L.	9	9	5	8
Cynodon dactylon (L.) Pers.	G	Cosmop.			A	Z		8	8	4	4
Cynoglossum cheirifolium L.	H	Steno-Medit.			A	Z		9	9	3	7
Cynoglossum columnae Ten.	T	NE-Medit.			A	Z		9	4	3	7
Cynoglossum creticum Mill.	H	Euri-Medit.			A	Z	sub Cynoglossum pictum Ait.	7	11	5	7
Cynosurus echinatus L.	T	Euri-Medit.			A			9	9	4	4
Cyperus distachyos All.	G	Subcosmop.				Z		7	10	7,5	5
Cyperus flavescens L.	T	Subcosmop.				Z		7,5	6	8	4
Cyperus fuscus L.	T	Paleotemp.			A			9	6	8	4

Cyperus longus L.	G	Paleotemp.			A			8	7	9	5,3
Cytinus hypocistis (L.) L.	G	Medit.			A			5	9,5	2,5	2
Cytinus ruber Fourr. ex Fritsch	G	W-Medit.			A			5	9,5	2,5	2
Cytisus infestus (C. Presl) Guss.	P	Steno-Medit.			A	Z	Zambrano indica Calicotome spinosa e C. villosa	7,5	8,6	3,5	3,8
Dactylis glomerata L. subsp. *glomerata*	H	Paleotemp.				Z	sub Dactylis glomerata L.	7	6	5	5
Dactyloctenium aegyptium (L.) Willd.	T	Paleotrop.		X (N)	A			9	9	3	7
Dactylorhiza maculata (L.) Soó subsp. *saccifera* (Brongn.) Diklić	G	Steno-Medit.				Z	sub Orchis maculata L.	7,1	3	7	2,5
Daphne gnidium L.	P	Steno-Medit.			A			7,3	8,3	3	3,3
Dasypyrum villosum (L.) P. Candargy	T	Medit.-Turan.			A	Z	sub Triticum villosum (L.) M. Bieb.	8	9,5	2	4
Datura stramonium L.	T	Cosmop.			A	Z		9	9	4	6
Daucus carota L.	H	Paleotemp.			A	Z		8,1	11	3	3
Delphinium halteratum Sm.	T	Steno-Medit.			A			8	9	3	2
Delphinium longipes Moris	T	Endem. Sar.				Z	segnalazione dubbia	8	9	3	2
Digitaria ciliaris (Retz.) Koeler	T	Subtrop.		X (N)	A			7,6	6,2	3	4
Digitaria sanguinalis (L.) Scop.	T	Cosmop.			A	Z		7,6	6,2	3	4
Dioscorea communis (L.) Caddick & Wilkin	G	Euri-Medit.			A			5,9	8	5	6
Diplotaxis erucoides (L.) DC.	T	W-Medit.			A	Z		8	8,3	3	5
Dittrichia graveolens (L.) Greuter	T	Medit.-Turan.			A			8,5	8,4	3	7
Dittrichia viscosa (L.) Greuter	H	Euri-Medit.			A			8,3	9	5	9
Draba verna L. subsp. *verna*	T	Circumbor.			A			8,1	6	2	3
Dysphania ambrosioides (L.) Mosyakin & Clemants	H	Cosmop.		X (I)	A			8	8	5	5
Ecballium elaterium (L.) A. Rich.	T	Euri-Medit.			A			7,7	8,8	3	8
Echinochloa crus-galli (L.) P.Beauv.	T	Paleotrop.			A	Z		6	7,5	8	8
Echinophora tenuifolia L.	H	Medit.-Turan.			A			8,5	11	3	3
Echium italicum subsp. *siculum* (Lacaita) Greuter & Burdet	H	Endem. Sic.			A			8,4	7	3	6
Echium plantagineum L.	H	Euri-Medit.			A	Z		8,6	7	3	6
Echium vulgare subsp. *pustulatum* (Sm.) Bonnier & Layens	H	Europ.			A			8,5	7	3,5	4
Eleocharis palustris (L.) Roem. & Schult.	G	Subcosmop.				Z		8,2	5,3	10	6
Elymus repens (L.) Gould subsp. *repens*	G	Circumbor.				Z	sub Triticum repens L.	7,4	5,4	5	7

Ephedra fragilis Desf.	NP	Steno-Medit.	EN		A			9	8	3	2
Epilobium hirsutum L.	H	Paleotemp.				Z		7,3	8	8	7
Epilobium palustre L.	H	Circumbor.				Z	segnalazione dubbia non presente in Sicilia	7,4	5	8	2,5
Equisetum ramosissimum Desf.	G	Paleotemp.				Z		7,8	7	4	2
Equisetum telmateia Ehrh.	G	Circumbor.			A			5,9	7	7,8	5,4
Eragrostis minor Host	T	Subcosmop.			A			8	8	3	4
Eragrostis pectinacea (Michx.) Nees	T	Americ.		X (I)	A			8	8	3	4
Erica arborea L.	NP	Steno-Medit.			A	Z		7,5	8	3	3
Erica multiflora L. subsp. *multiflora*	NP	Steno-Medit.			A	Z	confusa con Erica sicula Spreng.	8,5	9	3	1
Erigeron bonariensis L.	T	Americ.		X (I)	A	Z		7,5	8	3	7
Erigeron canadensis L.	T	N-Americ.		X (I)	A			7,8	6	5	7
Erigeron sumatrensis Retz.	T	Americ.		X (I)	A			7,7	8	6	7
Erodium ciconium (L.) L'Hér.	H	Euri-Medit.			A			8,4	9	2	5
Erodium cicutarium (L.) L'Hér	T	Subcosmop.			A			8,1	6,5	4	3,5
Erodium laciniatum (Cav.) Willd.	T	Steno-Medit.			A			8,5	9	2	6
Erodium malacoides (L.) L'Hér.	H	Medit.			A			8,5	9	2	6
Eruca vesicaria (L.) Cav.	T	Steno-Medit.				Z	sub Eruca sativa DC.	7,9	8	3	5
Eryngium campestre L.	H	Euri-Medit.			A	Z		8,7	7	3	3
Eryngium triquetrum Vahal	H	SW-Medit.				Z		9	8	4	4
Eucalyptus camaldulensis Dehnh.	P	Australia		X (N)	A			8	9	2	2
Eudianthe coeli-rosa (L.) Fenzl ex Endl.	T	SW-Medit.			A			8,5	9	3	4,5
Eupatorium cannabinum L.	H	Paleotemp.			A			7,1	7	7	7
Euphorbia akenocarpa Guss.	H	SW-Medit.			A	Z		7	11	6	4
Euphorbia ceratocarpa Ten.	Ch	Endem. Ital.			A	Z		4	6	4	4
Euphorbia characias L.	NP	Steno-Medit.			A			7,1	7,7	2	5,1
Euphorbia exigua L.	T	Euri-Medit.			A			7,3	9	2	4
Euphorbia falcata L.	T	Medit.-Turan.			A			7,9	7	4	3
Euphorbia helioscopia L.	T	Cosmop.			A	Z		7,4	7	3	6
Euphorbia maculata L.	T	N-Americ.			A			8,7	8	2	4
Euphorbia peplis L.	T	Euri-Medit.				Z		8,7	7	5	7
Euphorbia peploides Gouan	T	Cosmop.			A			8,7	7	5	7

Euphorbia terracina L.	H	Steno-Medit.			A			8,7	9	2	5
Fallopia convolvulus (L.) Á.Löve	T	Circumbor.				Z	sub Polygonum convolvulus L.	7,2	7	4	5
Fedia graciliflora Fisch. & C.A. Mey.	T	Steno-Medit.			A	Z	sub fedia cornucopiae (L.) Gaertn.	7,5	9	2	5
Ferula communis L.	H	Steno-Medit.			A	Z		8	8	3	6
Festuca ambigua Le Gall	T	Subcosmop.				Z	sub Festuca ciliata Danthoine ex DC.	8,2	9	2	2
Festuca danthonii Asch. & Graebn.	T	Subcosmop.			A			8,2	9	2	2
Festuca fasciculata Forssk.	T	Medit.-Atl.(Euri-)			A			8,5	10	1	1
Festuca heterophylla Lam.	H	Europ.-Caucas.				Z		5	5	4	4
Festuca incurva (Gouan) Gutermann	T	Euri-Medit.			A			9	9	2	2,5
Festuca ligustica (All.) Bertol.	T	Steno-Medit.-Occid.				Z	sub Vulpia ligustica Link	8	9	2	3
Festuca myuros L.	T	Subcosmop.			A			8	9	2	2
Ficaria verna subsp. *ficariiformis* (F.W. Schultz) B. Walln.	G	Euri-Medit.			A	Z	Ranunculus ficaria L.	4	5	6	7
Ficus carica L.	P	Medit.-Turan.			A	Z		7	8,8	3,5	7
Filago arvensis L.	T	Euri-Medit.				Z		9	7,3	2	1
Filago pygmaea L.	T	Steno-Medit.			A			9	10	3	3
Filago pyramidata L.	T	Euri-Medit.			A			8	8,8	5	1
Foeniculum vulgare Mill. subsp. *vulgare*	H	S-Medit.			A	Z		9	8	3	7
Foeniculum vulgare subsp. *piperitum* (Ucria) Bég.	H	S-Medit.			A	Z	sub Foeniculum officinale all.	9	8	3	7
Freesia alba (G.L. Mey.) Gumbl.	G	S-Afric.		X (N)	A			9	9	2	4
Fumana thymifolia (L.) Spach ex Webb	Ch	Steno-Medit.			A			9	9	2	1
Fumaria capreolata L.	T	Euri-Medit.			A			7	9	3	6
Fumaria officinalis L. subsp. *officinalis*	T	Eurasiat.			A	Z		7	7	4	6
Fumaria parviflora Lam.	T	Medit.-Turan.				Z	sub Fumaria leucantha Viv.	8	8	2	5
Gagea apulica Peruzzi & J.-M. Tison	G	Steno-Medit.-Occid.	EN		A			8	11	3	3
Gagea lojaconoi Peruzzi	G	Steno-Medit.-Nordorient.	LC		A			8	11	3	3
Gagea trinervia (Viv.) Greuter	G	S-Medit.	NT		A			7	11	2	4
Galactites tomentosus Moench	H	Steno-Medit.			A	Z		8	8,5	4	7
Galium aparine L.	T	Eurasiat.			A	Z		6	5,7	4	5

Galium divaricatum Lam.	T	Steno-Medit.			A					9	8,8	2	2
Galium lucidum All.	T	Euri-Medit.				Z				8	6,8	3	2
Galium murale (L.) All.	T	Steno-Medit.			A					9	8	2	3
Gastridium phleoides (Nees & Meyen) C.E. Hubb. subsp. *phleoides*	T	Steno-Medit.	LC		A					8	11	4	4
Gastridium ventricosum (Gouan) Schinz & Thell.	T	Medit.-Atl.(Euri-)			A					8,5	8,8	2,9	2,5
Gaudinia fragilis (L.) P. Beauv.	T	Euri-Medit.			A	Z				8,1	8	4,4	6
Gelasia hirsuta (Gouan) Zaika, Sukhor. & N.Kilian	H	NW-Medit.				Z	sub Scorzonera hirsuta (Gouan) L.			8,7	6	4,3	2
Gelasia villosa (Scop.) Cass. subsp. *columnae* (Guss.) Bartolucci, Galasso & F.Conti	H	Endem. Ital.				Z				7,8	7,2	3,4	2,4
Geranium molle L.	H	Eurasiat.			A					7,3	6,1	3,8	5,1
Geranium purpureum Vill.	T	Euri-Medit.			A					7	8	4,3	5
Geranium robertianum L.	H	Eurasiat.			A					4	6	5,5	8
Geranium rotundifolium L.	T	Paleotemp.			A					7	8	3,7	3
Gladiolus italicus Mill.	G	Euri-Medit.			A	Z	sub Gladiolus segetum Ker Gawl.			8,3	9	4,1	2
Glebionis coronaria (L.) Spach	T	Steno-Medit.			A	Z	sub Crysanthemum coronarium L.			7	9	4	8
Glebionis segetum (L.) Fourr.	T	Euri-Medit.			A	Z	sub Crysanthemum segetum L.			7	6	4	5
Globularia alypum L.	Ch	Steno-Medit.			A					8	9	1,7	3
Groenlandia densa (L.) Fourr.	I	Eurosib.				Z	sub Potamogeton densus L.			7	7	12	7
Gypsophila arrostoi Guss. subsp. *arrostoi*	Ch	Endem. Ital.			A					9	8	3	2
Hedera helix L.	P	Subatl.			A					4	5	5,3	7
Hedypnois rhagadioloides (L.) F.W. Schmidt	T	Steno-Medit.			A					9	9	3,5	5,9
Helianthemum aegyptiacum (L.) Mill.	T	Medit. Turan.			A					9	12	2	3,5
Helianthemum lippii (L.) Dum. Cours.	NP	S-Medit.	NT		A					9	11	2	3,5
Helianthemum salicifolium (L.) Mill.	T	Euri-Medit.			A					9	9	2,7	2
Helianthemum sanguineum (Lag.) Lag.	T	SW-Medit.	CR		A					9	11	2	2
Helichrysum stoechas subsp. *barrelieri* (Ten.) Nyman	Ch	Steno-Medit.-Orient.			A					9	9	2,8	1
Heliotropium europaeum L.	T	Medit.-Turan.			A					9	8	3,4	7
Helminthotheca echioides (L.) Holub	T	Euri-Medit.				Z	sub Helminthia echioides Gaertn.			9	8	4	6
Helosciadium nodiflorum (L.) W.D.J. Koch	H	Euri-Medit.			A					7	8	10	6
Hermodactylus tuberosus (L.) Mill.	G	Steno-Medit.-	LC		A					7	7	3	5

		Sett.									
Herniaria hirsuta L.	H	Paleotemp.			A			9	6	4	2
Hippocrepis ciliata Willd.	T	Steno-Medit.			A			9	9	2	3
Hirschfeldia incana (L.) Lagr.-Foss.	H	Subatl.			A	Z	Sinapis incana L.	9	9	3	6
Hordeum bulbosum L.	H	Paleotrop.			A			8	10	4	4
Hordeum murinum subsp. *leporinum* (Link) Arcang.	T	Euri-Medit.			A	Z	sub Hordeum murinum L	8	8	5	6
Hordeum secalinum Schreb.	H	Euri-Medit.-Occid.			A			8	8	5	5
Hymenocarpos circinnatus (L.) Savi	H	Steno-Medit.			A			9	9	2	2
Hyoseris radiata L.	H	Steno-Medit.				Z		9	8,5	2	1,5
Hyoseris scabra L.	T	Steno-Medit.			A			9	9	3	2
Hyparrhenia hirta (L.) Stapf	H	Paleotrop.			A			9	12	2	3
Hypericum perfoliatum L.	H	Steno-Medit.			A			6	8	4	4
Hypericum perforatum L.	H	Cosmop.			A			7	8	3,9	3,9
Hypericum triquetrifolium Turra	H	Steno-Medit.-Orient.			A			8	8	3,5	5,5
Hypochaeris achyrophorus L.	T	Steno-Medit.			A			9	9	3	3,6
Hypochaeris glabra L.	T	Euri-Medit.			A			9	8	3,3	1,8
Hypochaeris radicata L.	H	Europ.-Caucas.			A			9	8	2	2,8
Ionopsidium albiflorum Durieu	T	SW-Medit.				Z		9	10	2	1
Iris germanica L.	G	Origine ignota		X	A			9	6	2	5
Isatis tinctoria L. subsp. *tinctoria*	H	S-Europ.-S-Siber.		X		Z	Isatis canenscens DC.	9	3	3	3
Isoëtes histrix Bory	G	Steno-Medit.			A			7	10	10	2,3
Jacobaea delphiniifolia (Vahl) Pelser & Veldkamp	T	SW-Medit.			A			7	8	3	2
Jacobaea erratica (Bertol.) Fourr.	H	Euri-Medit.				Z	sub Senecio erraticus Bert.	7	6	6	4
Jacobaea lycopifolia (Desf. ex Poir.) Greuter & B.Nord.	Ch	Endem. Ital.				Z	sub Senecio lycopifolius Desf.	9	10	3	2
Juncus acutus L.	H	Euri-Medit.				Z		9	8	8	3
Juncus capitatus Weigel	T	Atl.			A			8	10	8	1
Juncus compressus Jacq.	G	Eurasiat.				Z		8	7	5	5
Juncus conglomeratus L.	G	Eurosib.				Z		7	7	7,6	5
Juncus fontanesii J. Gay subsp. *fontanesii*	G	Paleosubtrop.			A			8	8	8	5

Juncus hybridus Brot.	T	Euri-Medit.			A			8	8	7	5
Juniperus turbinata Guss.	P	Steno-Medit.				Z	sub Juniperus phoenicea L.	9	8	2,7	2
Juno planifolia (Mill.) Asch.	G	S-Medit.	EN		A	Z	sub Iris scorpioides Desf.	8	8	3,5	2
Kickxia commutata (Bernh. ex Rchb.) Fritsch subsp. *commutata*	H	Steno-Medit.			A	Z	sub Linaria graeca (Bory & Chaub.) Chav.	8	7	4	4
Kundmannia sicula (L.) DC.	H	Steno-Medit.			A			7	9	3,7	7
Lactuca saligna L.	T	Medit.-Turan.				Z		9	7	4	4
Lactuca virosa L.	T	Medit.-Atl.				Z		9	7	3,4	6
Lagurus ovatus L. subsp. *ovatus*	T	Euri-Medit.			A	Z		8	9	3,2	5
Lamarckia aurea (L.) Moench	T	Medit.-Turan.			A			9	10	3,3	5
Lamium amplexicaule L.	T	Paleotemp.				Z		7	7	3,9	7
Lamium bifidum Cirillo	T	Steno-Medit.			A			7	8	4,3	6
Lamium flexuosum Ten.	H	NW-Medit.				Z	sub Lamium pubescens Sibth.	5	5	5	7
Lathyrus cicera L.	T	Medit.-Turan.				Z		8	8	3,5	5
Lathyrus sativus L.	T	Origine ignota				Z		9	9	3,5	6
Lathyrus sylvestris L.	H	Europ.			A			7	5	4	4
Launaea fragilis (Asso) Pau	Ch	Saharo-Sind.	LC		A			9	12	1	1
Lavandula stoechas L.	NP	Steno-Medit.			A			9	9	3,3	1
Leontodon hispidus L. subsp. *hispidus*	H	Europ.-Caucas.			A			8	4,4	4,5	3
Lepidium draba L. subsp. *draba*	G	Medit.-Turan.				Z		8	6	5	4
Limodorum abortivum (L.) Sw.	G	Euri-Medit.			A	Z		4	7	4,3	3
Linaria multicaulis subsp. *humilis* (Guss.) De Leon., Giardina & Zizza	T	Endem. Sic.	EN		A	Z	sub Linaria stricta (sm.) Guss.	9	11	2	2
Linaria pelisseriana (L.) Mill.	T	Euri-Medit.			A			9	9	3,3	2
Linaria reflexa (L.) Desf. subsp. *reflexa*	T	SW-Medit.			A	Z		7	8	3	3
Linaria triphylla (L.) Mill.	T	W-Medit.				Z		8	8	3	5
Linum strictum L.	T	Steno-Medit.			A			9	9	3	2
Linum trigynum L.	T	Euri-Medit.			A			9	9	3,8	2
Linum usitatissimum subsp. *angustifolium* (Huds.) Thell.	H	Euri-Medit.			A			7	7	3,6	5
Lobularia maritima (L.) Desv.	Ch	Steno-Medit.			A			8	9	3,1	4
Loeflingia hispanica L.	T	Medit.			A			9	11	1	1

Logfia gallica (L.) Cosson & Germ.	T	Euri-Medit.			A	Z	sub Filago gallica L	9	8	1,8	1
Lolium multiflorum Lam.	T	Euri-Medit.				Z	sub Lolium siculum Parl.	7	7	4,6	6
Lolium perenne L.	H	Circumbor.			A	Z		8	10	5	7
Lolium rigidum Gaudin subsp. *rigidum*	T	Medit.			A			8	8	3	7
Lolium temulentum L.	T	Subcosmop.				Z	sub Lolium arvense L.	7	7	4	6
Loncomelos narbonense (L.) Raf.	G	Euri-Medit.			A	Z	sub Ornithogalum narbonense L.	8	7	4	5
Loncomelos pyrenaicum (L.) L.D. Hrouda subsp. *pyrenaicum*	G	Euri-Medit.			A			5	5	6	5
Lonicera implexa Aiton	P	Steno-Medit.			A			7	9	4	4
Lotus angustissimus L.	T	Euri-Medit.			A			9	8	7	4
Lotus cytisoides L.	Ch	Steno-Medit.			A			9	10	1	4
Lotus edulis L.	T	Steno-Medit.			A	Z		9	8	4	4
Lotus hirsutus L.	Ch	Euri-Medit.			A			9	6	1	1
Lotus hispidus DC.	T	W-Medit.			A			9	6	1	1
Lotus parviflorus Desf.	T	Steno-Medit.-Occid.			A			8,5	10	6	5
Lotus rectus L.	Ch	Steno-Medit.			A			7	9	7	3
Lotus tetragonolobus L.	T	Steno-Medit.			A			7,7	6	4,7	6,7
Lupinus albus L. subsp. *albus*	T	E-Medit.		X (N)	A	Z	Lupinus termis	8,3	9	4,3	6,7
Lupinus angustifolius L.	T	Steno-Medit.			A			8,3	9	3	5,3
Lupinus gussoneanus J. Agardh	T	Steno-Medit.			A			8	9	4,3	6
Lycium europaeum L.	NP	Euri-Medit.			A	Z		8,7	7	4	6
Lygeum spartum L.	H	Medit.			A			8,5	11	2,5	5,5
Lysimachia linum-stellatum L.	T	Steno-Medit.			A			8,4	9	2,8	1,7
Lysimachia nardii Arrigoni	T	Steno-Medit.-Occid.			A			7,8	7	6,9	2,8
Lythrum junceum Banks & Sol.	H	Steno-Medit.			A	Z	sub Lythrum graefferi Ten.	7,5	8	7,5	5
Magydaris pastinacea (Lam.) Paol.	H	Steno-Medit.-Occid.			A			9	9	3	2
Malva cretica Cav.	T	Steno-Medit.			A	Z	Lavatera hispida (Malva olbia)prob. Confusa con M . Cretica	7,3	7	4	7
Malva parviflora L.	T	Euri-Medit.			A			7,5	8	3,8	7,8
Malva sylvestris L.	H	Eurasiat.			A	Z		8,1	6	3,8	7,8

Mandragora autumnalis Bertol.	H	Steno-Medit.			A	Z	sub Mandragora officinarum Bert.	7,5	9	3,5	7
Marcus-kochia ramosissima (Desf.) Al-Shehbaz	T	W-Medit.				Z	Malcolmia parviflora (DC.) DC.	9	10	2	5,5
Maresia nana (DC.) Batt.	T	Steno-Medit.			A			9	10	2	5,5
Marrubium vulgare L.	H	Cosmop.			A	Z		8,7	8	3,8	7,8
Matricaria chamomilla L.	T	Paleotemp.				Z		7,4	5	5,3	5,6
Medicago disciformis DC.	T	Steno-Medit.			A			8	9	2,5	5,5
Medicago intertexta (L.) Mill.	T	W-Medit.				Z	sub Medicago echinus DC.	8	9	5,2	6,5
Medicago minima (L.) L.	T	Euri-Medit.			A	Z	sub Medicago recta (Desf) Wills	8,7	7	2,8	2
Medicago murex Willd.	T	Steno-Medit.				Z		8	9	5	5
Medicago rigidula (L.) All.	T	Euri-Medit.			A			8,4	8	2,4	3
Medicago tornata (L.) Mill.	T	W-Medit.				Z	sub Medicago helix Willd.	9	9	3	4
Medicago truncatula Gaertn.	T	Steno-Medit.			A			8	8	3	5
Melica ciliata L. subsp. *ciliata*	H	Euri-Medit.			A			8,1	7	2,4	2,8
Melica ciliata subsp. *magnolii* (Godr. & Gren.) K. Richt.	H	Medit.-Turan.			A	Z	sub M. magnolii Godr.	8,1	7	2,4	2,8
Melica minuta L.	H	Steno-Medit.-Occid.			A			7,3	9	2,4	2,8
Melica uniflora Retz.	H	Paleotemp.				Z		3,8	5	5,3	5,1
Mentha aquatica L. subsp. *aquatica*	H	Paleotemp.				Z		7,1	5	8,8	5
Mentha arvensis L.	H	Circumbor.				Z		7,1	5	7,5	7
Mentha longifolia (L.) L.	H	Paleotemp.				Z	sub Mentha sylvestris L.	7,3	5	8	8
Mentha pulegium L.	H	Euri-Medit.			A			8,2	7	7,2	6
Mentha suaveolens Ehrh.	H	Euri-Medit.			A			7,9	8	7,3	6
Mercurialis annua L.	T	Paleotemp.			A	Z		7,5	7	7	4,5
Micromeria graeca (L.) Benth. ex Rchb.	Ch	Steno-Medit.	LC		A	Z	sub Saureja sicula Guss.	8,4	8	3	2
Milium vernale M. Bieb.	T	Medit.-Mont.				Z		3	5	6	7
Mirabilis jalapa L.	G	Avv.		X	A			8	10	3	7
Misopates orontium (L.) Raf.	T	Euri-Medit.				Z	sub Antirrhinum orontium L.	7,6	7	5	5
Moraea sisyrinchium (L.) Ker Gawl.	G	Steno-Medit.		X	A			7,8	8	6	3,5
Morus alba L.	P	E-Asiat.		X (N)	A			7,8	7	5	5
Muscari botryoides (L.) Mill. subsp. *botryoides*	G	Euri-Medit.				Z	Sub M. botryoides (L.) Mill. - non presente in Sicilia	6,8	5	5	3,5
Muscari commutatum Guss.	G	Medit.				Z		7,8	8,5	3	3

		Centro-or.									
Muscari comosum (L.) Mill.	G	Euri-Medit.				Z		7,3	8	3	5
Muscari racemosum (L.) Mill.	G	Submedit.				Z		7,5	7,7	2,5	2
Myosotis arvensis (L.) Hill	T	Eurasiat.			A			6,7	5,3	4,5	4
Myosotis congesta Shuttlew.	T	Steno-Medit.	CR		A		Azzaro et al 2021	8,5	9	6	3
Myosotis ramosissima Rochel	T	Eurasiat.			A	Z	sub Myosotis collina Ehrh.	8,5	5	2,5	3
Myosotis sylvatica Hoffm. subsp. *elongata* (Strobl) Grau	T	Endem. Ital.				Z	sub Myosotis sylvatica Hoff.	5,5	4,6	5,6	7
Myosurus minimus L.	T	Subcosm.				Z		8	5	6,8	3
Myrtus communis L.	P	Steno-Medit.			A	Z		7,3	9	4	5
Narcissus obsoletus (Haw.) Steud.	G	Steno-Medit.			A	Z	sub Narcissus cupanianus Guss.	8	7	4	4
Narcissus tazetta L.	G	Steno-Medit.			A	Z		7,3	8	5,5	5
Nasturtium officinale W.T. Aiton	H	Cosmop.			A	Z		7,1	4	10	7
Neatostema apulum (L.) I.M. Johnst.	T	Steno-Medit.			A	Z	sub Myosotis apula L.	8	11	2	4
Neotinea lactea (Poir.) R.M. Bateman, Pridgeon & M.W. Chase	G	Steno-Medit.	NT		A			8	9	3,3	3
Nepeta apulejii Ucria	H	SW-Medit.				Z		9	11	3	2
Nigella damascena L.	T	Euri-Medit.			A	Z		7,9	9	4	4
Odontites bocconei (Guss.) Walp.	Ch	Endem. Sic.				Z	sub Odontites biancae Guss.	7	5	4	2
Oenanthe pimpinelloides L.	H	Medit.-Atl.(Euri-)			A			6,7	7	6	4
Olea europaea L.	P	Steno-Medit.			A	Z	sub Olea oleaster	8	10	3,3	5
Oloptum miliaceum (L.) Röser & H.R.Hamasha	H	Medit.-Turan.			A			6,2	9	4	5
Oncostema siculum (Tineo) Speta	G	Endem. Ital.				Z	sub Scilla sicula Tineo	7,8	9	4	3
Onobrychis caput-galli (L.) Lam.	T	Steno-Medit.			A			8	9	3,5	4
Ononis diffusa Ten.	T	S-Medit.			A			9	12	2,5	4
Ononis natrix subsp. *ramosissima* (Desf.) Batt.	H	Euri-Medit.			A			8,3	8	3,4	3
Ononis reclinata L.	T	Medit.-Turan.			A			8,4	9,5	1,8	4
Ononis sieberi DC.	T	NE-Medit.			A	Z	Zambrano indica Ononis pusilla (sub O. columneae)	8,5	7	2,9	2
Onopordum illyricum subsp. *cardunculus* (Boiss.) Arènes	H	Steno-Medit.			A			8,3	8	3,3	9
Ophrys apifera Huds.	G	Euri-Medit.				Z		7.6	6	4	2

Ophrys bertolonii Moretti subsp. *explanata* (Lojac.) Soca	G	Endem. Ital.	VU		A	Z	sub Ophrys bertolonii Moretti	8	8,7	4	3
Ophrys bombyliflora Link	G	Steno-Medit.-Occid.			A			7,7	9,3	3,7	3
Ophrys exaltata Ten. subsp. *exaltata*	G	Endem. Ital.	LC		A	Z		7,7	9,3	3,7	3
Ophrys forestieri (Rchb. f.) Lojac.	G	Steno-Medit.			A			8	8,7	3,4	3
Ophrys fusca Link subsp. *caesiella* (P.Delforge) Kreutz	G	Endem. Sic.				Z	sub Ophrys fusca Link	8	8,7	3,4	3
Ophrys *fusca* Link subsp. *pallida* (Raf.) E.G.Camus	G	SW-Medit.				Z	sub Ophrys pallida Link	8	8,7	3,4	3
Ophrys incubacea Bianca	G	Steno-Medit.			A	Z	sub Ophrys atrata L.	8	8	4	3
Ophrys lunulata Parl.	G	Endem. Sic.			A	Z		8	10	3	3
Ophrys lutea Cav.	G	Steno-Medit.	LC		A	Z		7,8	9,1	3,5	4
Ophrys oxyrrhynchos Tod. subsp. *oxyrrhynchos*	G	Endem. Ital.	LC		A			8	8	4	3
Ophrys passionis Sennen ex Devillers-Tersch. & Devillers subsp. *passionis*	G	Subendem.			A			8	8	4	3
Ophrys sicula Tineo	G	Euri-Medit.	LC		A			8	8	4	3
Ophrys speculum Link	G	Steno-Medit.-Occid.	LC		A			7,5	9,3	3,5	4
Ophrys sphegodes Mill. subsp. *panormitana* (Tod.) E.Nelson	H	S-Medit.			A			8	11	2	5,5
Ophrys sphegodes Mill. subsp. *panormitana* (Tod.) E.Nelson	G	Endem. Ital.	LC		A			8	8	4	3
Ophrys sphegodes Mill. subsp. *sphegodes*	G	Steno-Medit.				Z	sub Ophrys aranifera Hudson	8	8	4	3
Ophrys subfusca (Rchb.f.) Hausskn. subsp. *archimedea* (P.Delforge & M.Walravens) Kreutz	G	Endem. Sic.	LC		A			7.6	6	4	2
Ophrys tenthredinifera Willd. subsp. *grandiflora* (Ten.) Kreutz	G	Endem. Ital.	LC		A	Z	sub Ophrys tenthredinifera	8	8,8	2,8	4
Opopanax chironium (L.) W.D.J. Koch	H	Steno-Medit.			A			7	11	4	3
Opuntia ficus-indica (L.) Mill.	P	Neotrop.		X (I)	A	Z		9	11	2	2
Orchis anthropophora (L.) All.	G	Steno-Medit.			A	Z	sub Aceras anthropophorum (L.) R. Br.	7,4	6	3,4	3
Orchis italica Poir.	G	Steno-Medit.	LC		A	Z	sub Orchis undulatifolia Biv.	7,5	10	3,5	4
Origanum vulgare L. subsp. *viridulum* (Martrin-Donos) Nyman	H	S-Medit.			A	Z	sub Origanum virens Hoffmanns et Link	7	9	3,6	4
Ornithogalum collinum Guss.	G	S-Europ.	LC		A			7	8,3	5	3
Ornithogalum exscapum Ten.	G	Endem. Ital.				Z		8,5	8,5	3	5,5
Ornithogalum gussonei Ten.	G	Steno-Medit.	LC		A			7,4	8	3,5	4,3
Ornithogalum montanum Ten.	G	NE-Medit.			A			7	7	3,5	4,5

Ornithopus compressus L.	T	E-Medit.			A	Z		8,3	9	3	3,3
Orobanche alba Stephan ex Willd.	T	Orof. Eurasiat.				Z	sub Orobanche alexandri Tin.	7,6	8	3,8	5
Orobanche amethystea Thuill.	T	Subatl.			A			8	7,6	1,8	2
Orobanche crenata Forssk.	T	Medit.-Turan.			A			7,6	6,9	4,2	6,3
Orobanche variegata Wallr.	T	Steno-Medit.			A	Z	sub Orobanche spartii Vauch.	8	7	4	4,5
Osmunda regalis L.	G	Paleotrop.				Z		5,3	6	8,6	4,4
Osyris alba L.	NP	Euri-Medit.			A			7,5	9	3	3,8
Oxalis corniculata L.	Ch	Cosmop.			A			7,1	7,8	4,1	6
Oxalis pes-caprae L.	G	Africana		X (I)	A			7,3	9,5	3,8	6
Pallenis spinosa (L.) Cass.	H	Euri-Medit.			A			8,5	8,8	2,8	4,5
Papaver rhoeas L.	T	E-Medit.			A	Z		7,1	9	4,4	5,9
Parentucellia latifolia (L.) Caruel	T	Euri-Medit.			A			8	8,8	3	3,7
Parietaria judaica L.	H	Euri-Medit.			A	Z	sub Parietaria diffusa Mert. & K.	6,8	8	7	6,3
Parietaria officinalis L.	H	S-Europ.				Z		4,8	7	5,8	7
Paronychia argentea Lam.	H	Steno-Medit.			A	Z		8,3	9,3	2,7	3,5
Paronychia echinulata Chater	T	Steno-Medit.			A			8	9	3	3
Paspalum distichum L.	G	Subcosmop.		X (I)	A			7	8	8,7	6
Persicaria maculosa Gray	T	Subcosmop.			A			6,9	5	5	7
Petrorhagia dubia (Raf.) G. López & Romo	T	S-Medit.			A			8	8	2,5	3
Petrorhagia prolifera (L.) P.W. Ball & Heywood	T	Euri-Medit.			A			8,1	5	2,6	2,2
Petrorhagia saxifraga (L.) Link subsp. *gasparrinii* (Guss.) Pignatti ex Greuter & Burdet	H	Euri-Medit.			A			8,9	8	2,5	1,6
Phagnalon rupestre (L.) DC. subsp. *rupestre*	Ch	SW-Medit.			A			7,8	9	1,8	1
Phagnalon saxatile (L.) Cass.	Ch	Steno-Medit.			A			8,3	9	2,7	2
Phalaris aquatica L.	H	Steno-Medit.			A	Z	sub Phalaris nodosa Sibth.	7,7	7	4,7	4
Phalaris coerulescens Desf.	H	Steno-Medit.				Z		8	6	5	6
Phalaris minor Retz.	T	Paleosubtrop.			A			7,7	7	4,7	5
Phalaris truncata Guss. ex Bertol.	H	S-Medit.				Z		7,5	7	4,5	6
Phedimus stellatus (L.) Raf.	T	Steno-Medit.			A			5	9	5	4
Phelipanche nana (Reut.) Soják	T	Paleotemp.			A			7	6	3,7	4
Phelipanche ramosa (L.) Pomel	T	Paleosubtrop.				Z	sub Orobanche ramosa L.	6,8	6	5	6,4

Phillyrea latifolia L.	P	Steno-Medit.			A			8	8	3,6	5
Phlomis fruticosa L.	NP	Steno-Medit.-Sett.				Z		8,3	9	3,3	4
Phlomis herba-venti L.	H	Steno-Medit.			A	Z		8,3	9	3,5	5
Phragmites australis (Cav.) Trin. ex Steud.	G	Subcosmop.			A			7,2	5	9,7	5
Pimpinella gussonei (C.Presl) Bertol.	H	Endem. Sic.				Z		9	8	3	2
Pimpinella peregrina L.	H	Euri-Medit.			A			6,7	8	4	7
Pinus halepensis Mill. subsp. *halepensis*	P	Steno-Medit.			A			8	10	2,7	2
Pinus pinea L.	P	Euri-Medit.			A			8,5	8	2,5	3
Pistacia lentiscus L.	P	S-Medit.			A	Z		7,7	10	3	4
Plantago afra L.	T	Steno-Medit.			A	Z	sub P. psyllium L.	8	6	3	4
Plantago albicans L.	Ch	S-Medit.			A			8,3	11	2	3
Plantago bellardii All. subsp. *bellardii*	T	S-Medit.			A			8,3	10	3,5	2
Plantago coronopus L.	T	Euri-Medit.			A			8,1	7	5	3,5
Plantago lagopus L.	T	S-Medit.			A	Z		8	9	3	5
Plantago lanceolata L.	H	Cosmop.			A	Z		7,1	7	3	5
Plantago major L.	H	Eurasiat.			A			7,3	6	5	7
Plantago serraria L.	H	Steno-Medit.			A	Z		9	10	2	1
Platanus orientalis L.	P					Z		7,7	10	8	4
Poa annua L.	T	Cosmop.			A	Z		7	5,3	6	8
Poa bulbosa L. subsp. *bulbosa*	H	Paleotemp.			A	Z		9	6,8	2	1
Poa infirma Kunth	T	Euri-Medit.			A			8,2	7,8	7	5
Poa sylvicola Guss.	H	Euri-Medit.			A			9	8	6	6
Polycarpon tetraphyllum subsp. *diphyllum* (Cav.) O. Bolòs & Font Quer	T	Steno-Medit.			A			8,6	8	3,7	5
Polygala monspeliaca L.	T	Steno-Medit.			A			8,3	8,8	5	2
Polygonum arenastrum Boreau	T	Subcosmop.			A			7,6	9	4	6,3
Polygonum aviculare L.	T	Cosmop.			A	Z		7,6	9	4	6,3
Polypodium vulgare L.	H	Circumbor.				Z		5,5	5	5	2,8
Polypogon monspeliensis (L.) Desf.	T	Paleosubtrop.			A			8	8,8	9	6
Polypogon viridis (Gouan) Breistr.	H	Paleotrop.			A			7,8	8	8	6
Populus alba L.	P	Paleotemp.			A	Z		6	8	5	6

Populus nigra L.	P	Paleotemp.			A			6,1	7	8	7
Portulaca oleracea L.	T	Subcosmop.		X	A			7,5	8	4	7
Potamogeton natans L.	I	Subcosmop.				Z	sub Potamogeton fluitans non Roth	6,4	4,5	12	4
Potentilla reptans L.	H	Paleotemp.				Z		6,6	6	6	5
Poterium sanguisorba L.	H	Paleotemp.			A			7,4	6	3	2
Primula vulgaris Huds. subsp. *vulgaris*	H	Europ.			A			5,7	5	5	5
Prospero autumnale (L.) Speta	G	Euri-Medit.			A			8,4	8	2	3
Prospero obtusifolium (Poir.) Speta subsp. *intermedium* (Guss.) Soldano & F.Conti	G	SW-Medit.				Z	Scilla intermedia Guss.	8	11	3	2
Pseudopodospermum undulatum subsp. *deliciosum* (Guss.) Bartolucci, Galasso & F. Conti	G	SW-Medit.			A			8	8	3	3
Pseudoturritis turrita (L.) Al-Shehbaz	H	S-Europ.				Z	sub Arabis turrita L.	5,9	6,7	6	3
Pteridium aquilinum (L.) Kuhn subsp. *aquilinum*	G	Cosmop.				Z	sub Pteris aquilina L.	5	5	6	3
Pteris cretica L.	H	Pantrop.				Z		6	8	4	2
Puccinellia fasciculata (Torr.) E.P.Bicknell	H	Medit.-Atl.				Z	sub Glyceria permixta Guss.	8	7	6	7
Pulicaria dysenterica (L.) Bernh.	H	Euri-Medit.			A			7,9	6	7	5
Pulicaria odora (L.) Rchb.	H	Euri-Medit.			A			7	11	5	4
Pyrus communis subsp. *pyraster* (L.) Ehrh.	P	Eurasiat.			A			6,1	6,5	5,5	5
Quercus amplifolia Guss.	P	Pontica			A	Z	sub Quercus robur non L.	7	8	5	4,5
Quercus calliprinos Webb.	P	Steno-Medit.			A	Z		7	10	4	3,5
Quercus ilex L.	P	Steno-Medit.			A	Z		7	9	4	5
Quercus suber L.	P	Steno-Medit.			A	Z		8	8	3	3
Quercus virgiliana (Ten.) Ten.	P	Pontica			A	Z	sub Quercus robur non L.	7	8	5	4,5
Ranunculus bullatus L.	H	Steno-Medit.			A			7	8	3	3
Ranunculus millefoliatus Vahl	H	Medit. Mont.				Z		8	7	6	3
Ranunculus paludosus Poir.	H	Medit.-Turan.			A			9	9	3	4
Ranunculus pratensis C. Presl	H	Endem. Ital.	LC		A			7	8	4	5
Ranunculus repens L.	H	Paleotemp.				Z		6	5	7	7
Ranunculus repens L.	H	Paleotemp.				Z		6	5	7	7
Ranunculus velutinus Ten.	H	N-Medit.				Z		6	8	7	5
Reichardia intermedia (Sch. Bip.) Samp.	T	Steno-Medit.			A			7	11	2	6

Reichardia picroides (L.) Roth	H	Steno-Medit.			A	Z	sub Picridium vulgare Desf.	7,5	9,3	3	4
Reseda alba L.	H	Steno-Medit.			A			8	9	3,7	4
Reseda lutea L.	H	Europ.			A	Z	Reseda mucronulata L.	7,6	9	3,2	5
Reseda luteola L.	H	Circumbor.				Z	Reseda crispata L.	7,9	9	3,4	4
Rhamnus alaternus L.	P	Steno-Medit.			A			7,8	9,2	3,5	3
Rhus coriaria L.	P	S-Medit.		X	A			8,3	7,7	4,4	4
Robinia pseudoacacia L.	P	N-Americ.		X (I)	A			5,9	6	4,4	8
Romulea bulbocodium (L.) Sebast. & Mauri	G	Steno-Medit.			A			8,3	6	4,5	3
Romulea rollii Parl.	G	Steno-Medit.-Occid.			A			8,3	8,8	4,7	2,5
Rosa canina L.	NP	Paleotemp.				Z		7,5	7	4	4
Rosa sempervirens L.	NP	Steno-Medit.			A	Z		6,8	9	3,8	6
Rostraria cristata (L.) Tzvelev	T	Paleotemp.			A			8	5	3,2	3
Rubia peregrina L.	P	Steno-Medit.			A	Z		6	11	3,7	3,2
Rubus idaeus L.	NP	Circumbor.				Z		6,7	4	5,5	8
Rubus ulmifolius Schott	NP	Euri-Medit.			A	Z	sub Rubus dalmaticus Tratt.	7,8	8	4,5	7
Rumex acetosella L. subsp. *multifidus* (L.) Schübl. & G.Martens	H	Subcosmop.				Z	sub rumex multifidus L.	8	5	3,7	2
Rumex bucephalophorus L.	T	Medit.			A			8,3	12	2,8	4
Rumex conglomeratus Murray	H	Eurasiat.			A	Z		7,9	7	7	8
Rumex crispus L.	H	Subcosmop.				Z	sub Rumex elongatus Guss.	7,4	5	6	5
Rumex pulcher L. subsp. *pulcher*	H	Euri-Medit.			A			7,9	8	7	8
Rumex thyrsoides Desf.	H	W-Medit.			A			8	8	3	5
Ruscus aculeatus L.	Ch	Euri-Medit.			A	Z		4	8	4	5
Sagina apetala Ard.	T	Euri-Medit.			A			8	7	6	5
Salix alba L.	P	Paleotemp.			A	Z	sub Salix fragilis non L.	5	6	7	7
Salix pedicellata Desf.	P	Steno-Medit.			A	Z		8	8	7	5
Salsola oppositifolia Desf.	Ch	S-Medit.	EN		A			9	11	8	7
Salsola tragus L.	T	Paleotemp.			A			9	7	4	8
Salvia argentea L.	H	Steno-Medit.				Z		7	6	3	3
Salvia clandestina L.	H	SE-Europ.			A	Z		8	8	3	7
Salvia rosmarinus Spenn.	NP	Steno-Medit.			A	Z		9	8	2	2

Salvia verbenaca L.	H	Euri-Medit.			A			8	8	3	7
Sambucus nigra L.	P	Europ.			A	Z		7	6	5	9
Samolus valerandi L.	H	Subcosmop.	LC		A			7	6	8	6
Saponaria officinalis L.	H	Eurasiat.				Z		7	6	5	5
Scandix pecten-veneris L.	T	Euri-Medit.				Z		7	7	3	4
Schenkia spicata (L.) G. Mans.	T	Euri-Medit.			A			9	9	7	3
Scirpoides holoschoenus (L.) Soják	G	Euri-Medit.			A			8	8,5	8	4
Scolymus grandiflorus Desf.	H	SW-Medit.			A			9	10	2	5
Scolymus hispanicus L.	H	Euri-Medit.			A	Z		9	10	2	5
Scolymus maculatus L.	T	S-Medit.				Z		9	11	3	5
Scorpiurus subvillosus L.	T	Euri-Medit.			A			7	8,7	3	4
Scrophularia peregrina L.	T	Steno-Medit.			A	Z		5	9	4	5
Scrophularia vernalis L.	H	Europ.-Caucas.				Z		5	5,8	5,4	4
Sedum hispanicum L.	T	Pontica			A			9	6	2,7	2
Selaginella denticulata (L.) Spring	Ch	Steno-Medit.			A			5	8	4,3	1
Senecio glaucus subsp. *hyblaeus* Brullo	T	Endem. Sic.	LC		A			9	12	1	1
Senecio leucanthemifolius Poir. subsp. *leucanthemifolius*	T	Steno-Medit.	LC		A			9	9,5	3,6	2
Senecio vulgaris L. subsp. *vulgaris*	T	Cosmop.			A	Z		7	6	4,5	8
Serapias cordigera L.	G	Steno-Medit.			A	Z		9	8	4,3	2
Serapias lingua L.	G	Steno-Medit.-Occid.	LC		A	Z		9	8	3,8	2
Serapias parviflora Parl.	G	Steno-Medit.-Occid.			A			9	11	3,8	2
Serapias vomeracea (Burm. f.) Briq.	G	Euri-Medit.			A			9	8,5	4,4	2
Sherardia arvensis L.	T	Euri-Medit.			A	Z	sub sherardia cretica L.	8	6	4,1	5
Silene colorata Poir.	T	Steno-Medit.			A	Z	Zambrano indica Silene sericea non presente in gran parte del'italia	9	9	3	4
Silene gallica L.	T	Euri-Medit.			A			7,5	8,5	3,7	3
Silene latifolia Poir.	H	Steno-Medit.			A			7	9	3,7	6,5
Silene nocturna L. subsp. nocturna	T	S-Medit.			A			7	9	2,8	4
Silene vulgaris (Moench) Garcke subsp. *tenoreana* (Colla) Soldano & F.Conti	H	Paleotemp.			A	Z	Zambrano indica Silene inflata non presente in gran parte del'italia	8	6	4	2

Silybum marianum (L.) Gaertn.	H	Medit.-Turan.			A			9	10	3,7	7
Sinapis alba L.	T	E-Medit.	LC	X	A	Z		8	10	4,3	5
Sixalix atropurpurea (L.) Greuter & Burdet	H	Steno-Medit.		X	A	Z	sub Scabiosa cupanii Guss.	8	8	3,5	4
Smilax aspera L.	G	Subtrop			A			6	10	3,8	4
Smyrnium olusatrum L.	H	Medit.-Atl.(Euri-)			A			6	8	4,2	9
Smyrnium perfoliatum L.	H	Euri-Medit.				Z		5	8	4,7	7
Solanum dulcamara L.	NP	Paleotemp.				Z		7	5	8,6	8
Solanum linnaeanum Hepper & P.-M.L. Jaeger	NP	Africana		X (I)	A	Z	sub Solanum sodomaeum L.	9	11	1	4
Solanum nigrum L.	T	Cosmop.			A	Z		7	6	3	7
Solanum villosum Mill.	T	Euri-Medit.			A			7	6	5	7
Sonchus asper (L.) Hill subsp. *asper*	H	Cosmop.			A	Z		7	5	4	7
Sonchus bulbosus (L.) N.Kilian & Greuter subsp. *bulbosus*	H	Steno-Medit.				Z	sub Hieracium bulbosum L.	7	8	3	4
Sonchus oleraceus L.	H	Cosmop.			A	Z		7	5	4	8
Sorghum halepense (L.) Pers.	G	Cosmop.		X (I)	A	Z		8	7	6	7
Sparganium erectum L.	I	Eurasiat.				Z	Sub Sparganium ramosum Huds.	7	5	10	5
Spartium junceum L.	P	Euri-Medit.			A	Z		8,3	8	4,3	3
Spergularia marina (L.) Besser	T	Subcosmop.			A			7,6	7	6,8	5
Squilla maritima (L.) Steinh.	G	Steno-Medit.				Z		9	8	4	3
Stachys arenaria Vahl	H	W-Medit.	CR		A			9	8	3	2
Stachys germanica L. subsp. *dasyanthes* (Raf.) Arcang.	H	Endem. Sic.				Z	sub Stachys dasyanthes Raf.	7,1	6	7	8
Stachys major (L.) Bartolucci & Peruzzi	Ch	Steno-Medit.			A	Z	sub Prasium majus L.	8,5	10	3	5
Stachys romana (L.) E.H.L. Krause	T	Steno-Medit.			A			8	9	3,5	3
Stellaria media (L.) Vill.	H	Cosmop.			A			6,7	7	5	8
Sternbergia lutea (L.) Ker Gawl. ex Spreng.	G	Medit. Mont.				Z		7,3	6	4	4
Stipa letourneuxii Trabut subsp. *letourneuxii*	H	S-Medit.			A	Z	sub Stipa gigantea Link	7,9	8,8	3,2	4,2
Stipellula capensis (Thunb.) Röser & H.R.Hamasha	T	Steno-Medit.			A			9	11	1	6
Sulla coronaria (L.) Medik.	H	W-Medit.			A	Z	sub Hedisarum coronarium	8	9	5	5
Sulla spinosissima (L.) B.H. Choi & H. Ohashi	T	W-Medit.			A			8,5	9,8	2,5	2
Symphytum bulbosum K.F.Schimp.	G	S-Europ.				Z		5	6,6	5	3
Tamarix africana Poir.	P	W-Medit.			A	Z		8,5	9,5	6	3

Taraxacum minimum (V.Brig.) N.Terracc.	H	Euri-Medit.				Z	presenza dubbia in Sicilia	7,7	7,5	4	6
Teucrium capitatum L.	Ch	Steno-Medit.			A			8,5	11	2,9	2,1
Teucrium creticum L.	NP	S-Medit.				Z		9	11	1	2
Teucrium flavum L.	Ch	Steno-Medit.			A			8,3	8	3	2,7
Teucrium fruticans L.	NP	Steno-Medit.			A	Z		8,3	8	3	3
Teucrium luteum (Mill.) Degen	Ch	Steno-Medit.			A	Z	sub Teucrium polium L.	9	11	1	2
Thalictrum calabricum Spreng.	H	Endem. Ital.				Z		5	7	6	6
Thapsia asclepium L.	H	Steno-Medit.			A			8	8	3	7
Theligonum cynocrambe L.	T	Steno-Medit.			A			6,7	9	4,3	6,3
Thesium humile Vahl	T	Medit.-Atl.(Euri-)			A			8,5	9	2,5	3,5
Thymbra capitata (L.) Cav.	Ch	Steno-Medit.	NT		A	Z	sud thymus serphyllum auct. non L.	8,5	10	2,5	2
Thymelaea hirsuta (L.) Endl.	Ch	S-Medit.			A			8,3	8	4	5
Tolpis umbellata Bertol.	T	Steno-Medit.			A	Z	sub Tolpis barbata (L.) Gaertn	9	9	2	1
Tolpis virgata (Desf.) Bertol. subsp. *grandiflora* (Ten.) Arcang.	H	Endem. Ital.				Z	sub Tolpis grandiflora Ten.	8,3	8	4	2
Tolpis virgata (Desf.) Bertol. subsp. *virgata*	H	Steno-Medit.				Z	sub Tolpis quadriaristata Biv.	8,3	8	4	2
Tordylium apulum L	T	Steno-Medit.			A			8	9	3,3	6
Torilis nodosa (L.) Gaertn. subsp. *nodosa*	T	Euri-Medit.			A			7,8	8	4	6,3
Tragopogon cupanii Guss. ex DC.	T	Endem. It.				Z		8,1	9	3	5,3
Tragopogon porrifolius L.	H	Euri-Medit.			A	Z		8,1	9	3	5,3
Tribulus terrestris L.	T	Cosmop.			A			8,4	8	3	5
Trifoliium congestum Guss.	T	Steno-Medit.-Or.				Z		7,5	10	5,5	2
Trifolium angustifolium L.	T	Euri-Medit.			A			8,1	9,5	3,7	3
Trifolium arvense L.	T	Paleotemp.			A			8,2	5	2,9	2
Trifolium campestre Schreb.	T	Paleotemp.			A	Z		8	5,8	3,9	3
Trifolium cherleri L.	T	Euri-Medit.			A			8	9	4	3
Trifolium glomeratum L.	T	Euri-Medit.			A			8	7,7	3,3	2
Trifolium grandiflorum Schreb.	T	E-Medit.				Z	sub T. speciosum Willd.	6,5	6	4	4
Trifolium infamia-ponertii Greuter	T	N-Medit.			A			7	11	4	6
Trifolium nigrescens Viv. subsp. *nigrescens*	T	Euri-Medit.			A			7,5	6	4,5	6,5

Trifolium pallidum Waldst. & Kit.	H	Euri-Medit.-Orient.			A			7,8	8	5	3
Trifolium pratense L.	H	Eurosiber.				Z		7,3	5	5	6
Trifolium repens L.	H	Paleotemp.				Z		7,7	5	5	7
Trifolium resupinatum L.	H	Paleotemp.			A			8,1	8	6	5
Trifolium scabrum L.	T	Euri-Medit.			A			8,5	8	2	2
Trifolium stellatum L.	T	Euri-Medit.			A	Z		8	9	3	5
Trifolium subterraneum L. subsp. *subterraneum*	T	Euri-Medit.			A			8,4	9	3	4
Trifolium suffocatum L.	T	Steno-Medit.-Occid.			A			8	9	4	2
Trifolium tomentosum L.	T	Paleotemp.			A			8	9	4	4
Trigonella esculenta Willd.	T					Z	sub T.corniculata, forse confusa con T. sulcata	9	9	4	5
Trigonella foenum-graecum L.	T	W-Asiatica		X (N)	A			8,5	9	3	4
Trigonella infesta (Guss.) Coulot & Rabaute	T					Z	sub Melilotus infesta Guss.	8,5	9	3	6
Trigonella sulcata (Desf.) Coulot & Rabaute	T	S-Medit.			A			8	8	4	5
Trigonella wojciechowskii Coulot & Rabaute	T	Steno-Medit.				Z	sub Melilotus neapolitana Ten.	8	11	3	5
Tripodion tetraphyllum (L.) Fourr.	T	Steno-Medit.			A			8	10	2	4
Trisetaria aurea (Ten.) Banfi & Galasso	T	Steno-Medit.-Orient.	LC		A			8	9	2	5
Triticum turgidum subsp. *durum* (Desf.) Husn.	T	Avv.			A	Z	sub Triticum durum Desf.	8,5	8	5	5
Triticum vagans (Jord. & Fourr.) Greuter	T	Medit.-Turan.			A			8,5	8	5	5
Tuberaria guttata (L.) Fourr.	T	Euri-Medit.			A			8,8	10	2	1
Tuberaria villosissima (Pomel) Grosser	T	SW-Medit.	LC		A			8,8	10	2	1
Tussilago farfara L.	G	Paleotemp.				Z		9	5	6	7
Typha angustifolia L.	G	Circumbor.				Z		8,2	7	10	7
Ulex europaeus L.	P	Subatl.				Z	non presente in Sicilia	7,5	7	5	2
Ulmus minor subsp. *canescens* Bartolucci & Galasso	P	E-Medit.			A			5	7,5	4,5	2
Umbilicus horizontalis (Guss.) DC.	G	Steno-Medit.			A			5	8	3	4
Umbilicus rupestris (Salisb.) Dandy	G	Medit.-Atl.(Euri-)			A			5	8	3	3
Urospermum dalechampii (L.) F.W. Schmidt	H	Euri-Medit.-Occid.			A	Z		8	8	3	5
Urospermum picroides (L.) Scop. ex F.W. Schmidt	T	Euri-Medit.			A			9	9	2	6,3

Urtica dioica L.	H	Subcosmop.			A	Z		6	6	6	8,5
Urtica membranacea Poir.	T	S-Medit.			A	Z		7	9	5	7,3
Urtica pilulifera L.	T	S-Medit.			A			7	8	5	7,7
Urtica urens L.	T	Subcosmop.			A	Z		7	6	5	7,9
Vachellia karroo (Hayne) Banfi & Galasso	P	Africana		X (N)	A			9	12	2	2
Valantia muralis L.	T	Steno-Medit.			A			9	9	2	4,5
Valerianella eriocarpa Desv.	T	Steno-Medit.			A			9	9	3	2
Valerianella microcarpa Loisel.	T	Steno-Medit.			A			9	9	5	3
Verbascum sinuatum L.	H	Euri-Medit.			A	Z		9	10	3	8
Verbena officinalis L.	H	Cosmop.			A	Z		9	5	5	6
Veronica agrestis L.	T	Europ.				Z		5	6	6	7
Veronica anagallis-aquatica L.	H	Cosmop.			A			7	6	9	6
Veronica arvensis L.	T	Subcosmop.			A	Z		5	5	5	6
Veronica serpyllifolia L.	H	Circumbor.				Z		6	5	4	5
Viburnum tinus L.	P	Steno-Medit.				Z		5	9	4	3
Vicia hybrida L.	T	Euri-Medit.			A	Z		7	9	3	5
Vicia lathyroides L.	T	Euri-Medit.			A			8	7	2	2
Vicia sativa L.	T	Medit.		X	A	Z		9	9	5	5
Vicia serratifolia Jacq.	T	Euri-Medit.			A			7	8,5	3	2
Vicia villosa Roth	H	Steno-Medit.			A			7,1	8	4	5
Vinca major L.	H	Euri-Medit.				Z		5,5	8,5	4	3
Viola odorata L.	H	Euri-Medit.				Z		5	6	5	8
Vitis vinifera L. subsp. *sylvestris* (Gmel.) Hegi	P	Euri-Medit.				Z		6	8	6	6
Xanthium orientale L. subsp. *italicum* (Moretti) Greuter	T	S-Europ.		X (I)	A			8	7	5	6
Xanthium spinosum L.	T	S-Americ.		X (I)	A			9	10	2	1
Zannichellia palustris L.	I	Cosmop,				Z		6,3	8	12	6
